# Gene expression profiling-based risk prediction and profiles of immune infiltration in diffuse large B-cell lymphoma

**DOI:** 10.1038/s41408-020-00404-0

**Published:** 2021-01-07

**Authors:** Selin Merdan, Kritika Subramanian, Turgay Ayer, Johan Van Weyenbergh, Andres Chang, Jean L. Koff, Christopher Flowers

**Affiliations:** 1 Department of Industrial and Systems Engineering, Georgia Institute of Technology, Atlanta, GA USA; 2 Department of Molecular Imaging and Therapeutics, Weill Cornell Medicine, New York, NY USA; 3 Department of Microbiology, Immunology and Transplantation, Laboratory of Clinical and Epidemiological Virology, Rega Institute for Medical Research, KU Leuven, Leuven, Belgium; 4 Department of Hematology and Medical Oncology, Emory University School of Medicine, Atlanta, GA USA; 5 Department of Lymphoma and Myeloma, Division of Cancer Medicine, University of Texas MD Anderson Cancer Center, Houston, TX USA

**Keywords:** Translational research, Cancer microenvironment

## Abstract

The clinical risk stratification of diffuse large B-cell lymphoma (DLBCL) relies on the International Prognostic Index (IPI) for the identification of high-risk disease. Recent studies suggest that the immune microenvironment plays a role in treatment response prediction and survival in DLBCL. This study developed a risk prediction model and evaluated the model’s biological implications in association with the estimated profiles of immune infiltration. Gene-expression profiling of 718 patients with DLBCL was done, for which RNA sequencing data and clinical covariates were obtained from Reddy et al. (2017). Using unsupervised and supervised machine learning methods to identify survival-associated gene signatures, a multivariable model of survival was constructed. Tumor-infiltrating immune cell compositions were enumerated using CIBERSORT deconvolution analysis. A four gene-signature-based score was developed that separated patients into high- and low-risk groups. The combination of the gene-expression-based score with the IPI improved the discrimination on the validation and complete sets. The gene signatures were successfully validated with the deconvolution output. Correlating the deconvolution findings with the gene signatures and risk score, CD8+ T-cells and naïve CD4+ T-cells were associated with favorable prognosis. By analyzing the gene-expression data with a systematic approach, a risk prediction model that outperforms the existing risk assessment methods was developed and validated.

## Introduction

Diffuse large B-cell lymphoma (DLBCL) is the most common lymphoma in the United States, accounting for about one-third of adult cases of non-Hodgkin’s lymphoma^1^. Despite the high cure rates for DLBCL, outcomes remain varied in part due to heterogeneity in the disease at the clinical, pathological, and molecular levels^2–5^. The clinical risk stratification of DLBCL patients is currently assessed by the International Prognostic Index (IPI) scoring system, which was developed more than two decades ago and utilizes five clinical factors: age, performance status, stage, number of extranodal sites, and serum lactate dehydrogenase (LDH)^6^. However, IPI is sub-optimal in its identification of high-risk DLBCL patients as it does not differentiate low-risk and high-risk stratification groups, especially when considering response to first-line therapy^7^. To develop individualized treatment strategies, increasing efforts have been directed toward identifying prognostic factors for accurate risk stratification of patients with DLBCL^2,8,9^. Strategies involving mutation analyses and gene-expression profiling have been employed to subtype the malignant cells in the tumor. In particular, activated B-cell-like DLBCL and germinal center B-cell-like DLBCL, and subgroup clusters defined by tumor sequencing have been shown to carry prognostic significance^10^.

Emerging evidence highlights the important role of tumor microenvironment (TME) in cancer initiation, metastasis, progression, and response to therapeutic agents^11–13^. In hematological cancers, malignant cells may participate as part of the dysregulated immune milieu through altered secretion of those cytokines that normally keep proliferation in check. Understanding the types and roles of immune cells in the TME is therefore key to develop strategies aimed at targeting the tumor and improving patient outcomes. The immune response to cancer is characterized by numerous tumor-infiltrating immune cells which interact with each other. Novel therapies like anti-CD19 chimeric antigen receptor (CAR) T-cells and NK (natural killer) cells have taken advantage of this immune response to provide new treatment options for patients with relapsed DLBCL^14–16^. Characterization of the TME in previously untreated DLBCL could offer important insights into the complex relationship between certain immune cell types, paving the way for a more personalized approach to treatment planning in DLBCL.

Recently developed computational methods to estimate the relative proportions of immune cell types using gene-expression data profiled from tissues such as bulk tumors can aid in this effort^17^. CIBERSORT is a deconvolution approach that has been shown to outperform other existing methods in resolving closely related cell subsets, unknown mixture content, and noise^18^. In this study, we propose a comprehensive statistical framework designed to identify the best-performing prognostic model for the personalized risk prediction of DLBCL patients using genetic and clinical features from a large RNA-seq dataset. CIBERSORT was applied to profile the diversity and landscape of tumor-infiltrating immune cells in DLBCL and evaluate the relationship between immune cell populations and prognostic outcomes.

## Methods

### RNA-sequencing data analysis

The data from 775 preprocessed and aligned tumor RNA-seq transcriptomes published by Reddy et al.^2^ was used. RNA-seq data was collected from the fresh-frozen paraffin-embedded tumor block collected prior to initiation on a rituximab-containing standard regimen. Aligned read counts were subsequently summarized and quantified using featureCounts program^19^. The built-in human gene annotation of featureCounts was used as a reference genome assembly. Exons were grouped into genes and the read summarization was performed at the gene level. The quantification was not strand specific and paired ends were excluded from the quantification. The default method (union) was kept, which selects the gene with the strongest overlap if two genes are associated with a read. Gene IDs were annotated to gene symbols using MyGene^20^. Gene IDs without an associated symbol were removed from further analysis. Duplicate gene symbol entries were also filtered, favoring to keep the entry with the greatest read strength.

To reduce the potentially adverse effects of noise in statistical analyses, 35 samples with expression of fewer than 12,000 genes was omitted; 22 samples with unknown survival and censoring was also omitted. The remaining 718 patient cases were designated as the core set for the statistical analyses. Gene-expression measurements were normalized using the Trimmed Mean of M-values normalization method of edgeR package and the data was log_2_ normalized^21^. Differential expression analysis was performed with edgeR. To determine deferentially expressed genes, genes were identified according to a *p*-value cut-off of 5% and then applied a fold-change cut-off of 50% and then were selected. To interpret the differential expression results in biological context, gene ontology (GO) enrichment analysis using the *goana* function in edgeR with focus on the ontology of biological process was conducted. To identify the functions underlying these genes, pathway enrichment analysis was performed using the GOenrichmentAnalysis (GEA) method of the Weighted Correlation Network Analysis package (WGCNA)^22^. The WebGestalt (WEB-based Gene SeT AnaLysis Toolkit) tool for Kyoto Encyclopedia of Genes and Genomes (KEGG) pathway enrichment analyses of the gene signatures^23^ was used. The pathways with *p-*values *<* 0.05 (after FDR correction) were regarded as significantly enriched.

### Evaluation of tumor-infiltrating immune cells

In order to determine immune cell types and origin from the tumor transcriptomes, CIBERSORT analysis using the LM22 default reference was applied. Based on prior publications^17,24^, quantile normalization (QN) on RNA-seq data^25^ was disabled. The LM22 reference matrix was adapted to exclude B-cells from further study to represent the TME instead of the total sample/biopsy. The tumor immune infiltrate with and without B-cells, which we refer to as tumor and TME, respectively, was measured.

To assess the association between immune infiltration and prognosis, the relative proportions of the immune cell types within subgroups of DLBCL patients by various clinical traits and outcomes including age (≤60 vs. *>*60 years), sex (male vs. female), IPI (0–1 vs. 2 or more), the cell of origin classification (ABC vs. GCB), response to treatment, and survival outcome at 2 years from diagnosis was compared. The changes in the proportion of immune cell subtypes between groups were assessed by log_2_ fold-change, where the poorer prognostic factor was selected as the reference. In addition to these clinical risk groups, the changes in the immune infiltration across risk groups with distinct survival outcomes (alive or dead) at 2 and 5 years from diagnosis was also evaluated, where the fold-change was calculated as log_2_(base mean in alive*/*base mean in dead) for each cell subtype.

### Identification of gene-expression signatures

To identify gene-expression signatures associated with survival in DLBCL, the analytical approach previously described by Dave et al.^26^ was implemented. In this approach, samples in the core set are first randomly divided into two parts: a training set of 70% of the patients and a validation set of 30% of the patients, which were balanced with respect to the length of follow-up. Cox proportional hazards models to identify genes that were statistically associated with survival in the training set was used. The genes with expression levels associated with favorable and unfavorable prognosis were organized separately with hierarchical clustering algorithms to identify survival-associated signatures. Within each signature, member gene-expression levels were averaged to create a genetic-expression signature for each patient.

Hierarchical clustering procedures on the training test to detect gene signatures was implemented and evaluated the association between the gene signatures and survival in the training and testing sets. For hierarchical clustering, Pearson correlation to construct dissimilarity matrix and average linkage method to define the distance between clusters was used. Two different methods to detect gene clusters— (1) clusters are defined by cutting off branches using a constant cut-off value of dissimilarity (i.e., correlation) and (2) clusters are defined by Dynamic Tree Cut method^27^—were implemented. In the first method, which from this point on will be referred to as predefined cut-off clustering, each gene signature was defined as a cluster such that within each cluster genes have inter-cluster dissimilarities less than a predefined level of dissimilarity (*r* > 0.4). To overcome the inflexibility of the first method for cluster detection, the Dynamic Tree Cut method was implemented, which is a top-down approach that detects clusters through an iterative process of cluster decomposition and combination on a dendrogram^27^. The clustering methods are described in detail in the Supplementary Material. When defining the final set of gene-expression signatures, the gene signatures that were significant predictors of survival only in the training set and not in the testing set were excluded. The signatures of poor and good prognosis genes are referred to as “unfavorable” and “favorable”.

### Construction of a gene expression profiling-based survival predictor

To determine the best set of gene signatures for prognostic prediction, the Lasso method was implemented to identify the important gene signatures on the training set and developed multivariable Cox models using these signatures^28^. The Lasso method shrinks the regression coefficients toward zero by penalizing the size of the coefficients with *L*_1_ penalty term^29^. If the log partial likelihood

is denoted as *l*(*β*), the penalized log partial likelihood becomes $l\,(\beta ) - \lambda \mathop {\sum}\nolimits_{i = 1}^p {\left| {\beta _i} \right|}$, where *p* is the number of predictors^28^ and *λ* is the tuning parameter determining the amount of shrinkage. The Lasso variable was the chosen selection method as it helps to increase the model interpretability by eliminating irrelevant variables that are not associated with the response variable, and therefore, reduces over-fitting. To choose the tuning parameter *λ*, 10-fold cross-validation was performed on the training set. The optimal tuning parameter was defined as the value within one standard deviation of the minimum cross-validated partial likelihood deviance to obtain the most parsimonious model. The gene signatures with non-zero coefficients at the optimal cross-validated log-likelihood were referred as *survival signatures*. The set of survival signatures found using the predefined cut-off clustering method is referred to as *signature set 1* and the set of survival signatures detected by the Dynamic Cut Tree method as *signature set 2*.

Multivariable models of survival were developed using signature sets 1 and 2 on the training set and validated the prognostic ability of the multivariable models by calculating survival-predictor scores for the validation set cases using the coefficients of the Cox models estimated on the training set. Patients were stratified in the validation set based on their risk scores into high- and low-risk groups according to the optimal cut-off for the survival-predictor score determined by the survminer package^30^. Log-rank tests were used to determine whether there was a significant difference between the Kaplan-Meier survival curves for the resulting risk groups.

In addition to log-rank tests, the time-dependent area (AUC) under the receiver operator curve (ROC) was used to evaluate the prognostic accuracy of survival predictors^31^. Since the majority of adverse DLBCL events occur in the first 2 years after diagnosis^32^, the ability to accurately identify high-risk individuals could improve the selection of appropriate treatment for these patients. Therefore, the AUC of the time-dependent ROC was evaluated at time points of 2, 5, and 10 years from diagnosis. To assess the independence of the risk groups defined by the IPI and the outcome predicated on gene-expression profiles, multivariable Cox regression analysis was conducted.

To investigate whether certain tumor-infiltrating immune cell sub-populations significantly impact prognosis, the risk score was correlated derived from our gene-expression prediction model with the relative proportions of immune cell types enumerated by the CIBERSORT algorithm. The prognostic implications of this analysis were further confirmed by assessing the correlation between cell proportions and gene signatures.

## Results

### The landscape of immune infiltration in DLBCL

**Fig. 1:**
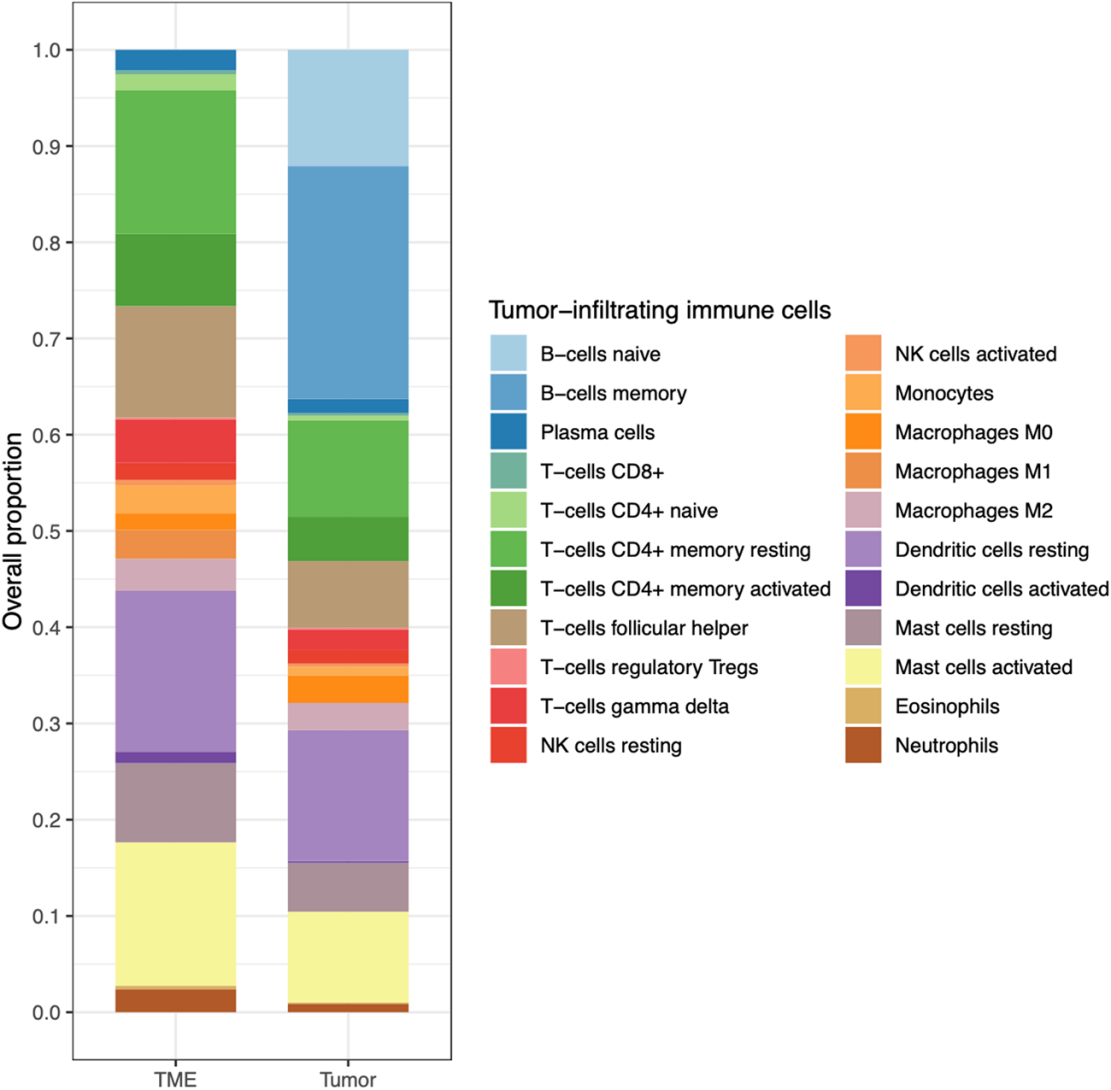
TME Immune Cell Content. The overall immune cell content within the TME and tumor.

Figure 1 shows the mean relative abundances of tumor-infiltrating immune cells estimated by the CIBERSORT algorithm in the TME, defined as tumor in the absence of B-cells, and tumor samples. B-cells were predicted to make up *>*30% of the entire tumor sample. Neutrophils, CD4 + naive T-cells, CD8 + T-cells, CD4 + memory resting cells, and M0 macrophages were notably visible in the TME.

### Deconvolution analyses stratified by clinical risk groups

**Fig. 2:**
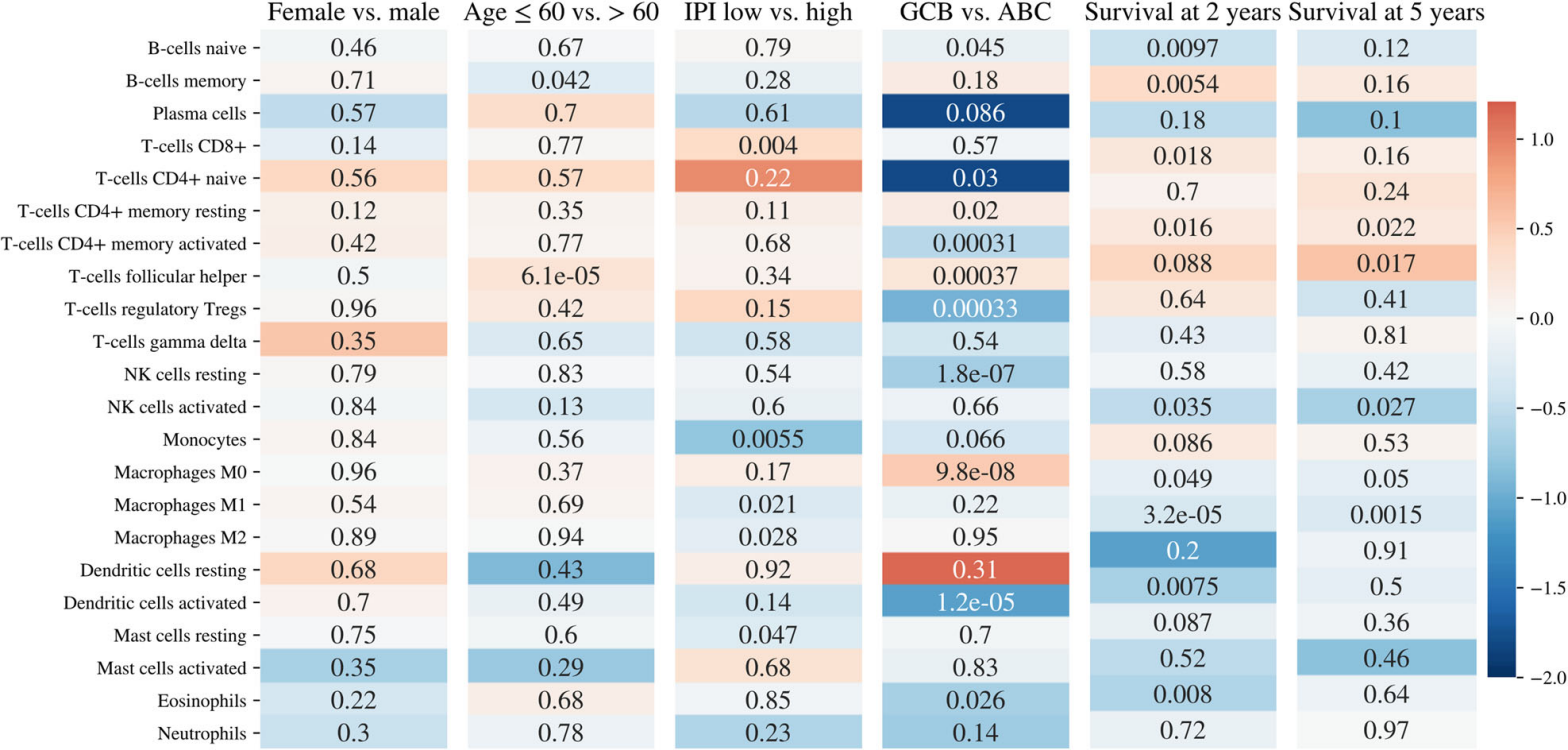
Clinically Stratified Immune Cell Type Assessment. Heatmaps showing the differential assessment of the immune contexture within clinical risk groups.

Heatmaps in Fig. 2 summarize differences in immune environment when cases were stratified by clinical features and overall survival. Favorable clinical features such as age <60 years, low IPI score, being female, and Germinal B-cell (GBC) subtype tended to show greater naive CD4 + T-cells, memory CD4 + T-cells, follicular helper T-cells, regulatory T-cells, CD8 + T-cells, and M0 macrophages. There was also a decrease in monocytes and M1-M2 Macrophages. Overall survival at 2 years similarly showed an increase in CD8 + T-cells and CD4 + T-cell subsets, while at 5 years there was an increase in follicular helper and regulatory T-cells. Overall, there was evidence of T-cell activation with favorable clinical features. This was consistent with findings from Reddy et al.^2^ where signatures, in the stromal and immune response groups, such as the regulatory T-cells were associated with improved survival outcomes. Follicular T-cells were not included in the analysis performed by Reddy et al.

### Survival signature analysis

The clinical characteristics of 718 patients with complete overall survival information are summarized in Table S1. A total of 1989 genes were identified by univariate Cox models as being associated with survival in the training set at a significance level of *p* < 0.1 using a Wald test. The genes associated with good prognosis (1139 genes) and poor prognosis (850 genes) in the training set were clustered separately via hierarchical clustering algorithms. The predefined cut-off clustering method identified six gene signatures among the genes predicting good prognosis and six signatures among the genes predicting poor prognosis (Table S2 and Figs. S1–S3). The Dynamic Cut Tree method identified 11 gene signatures among the genes predicting good prognosis and 10 signatures among the genes predicting poor prognosis (Tables S3, S4 and Figs. S4, S5). The gene-expression signatures were named on the basis of the association of each signature with survival (favorable/unfavorable). All the gene signatures detected by clustering the two methods were predictive of survival on the training set (all *p* < 0.005, Table S5). The three signatures detected by the predefined cut-off clustering method and seven signatures detected by the Dynamic Cut Tree method that were not significant predictors of survival on the testing set at the significance level of *p* < 0.05 were omitted from further analyses. The three signatures with 0.05 < *p* < 0.06 were kept on the testing set.

**Fig. 3:**
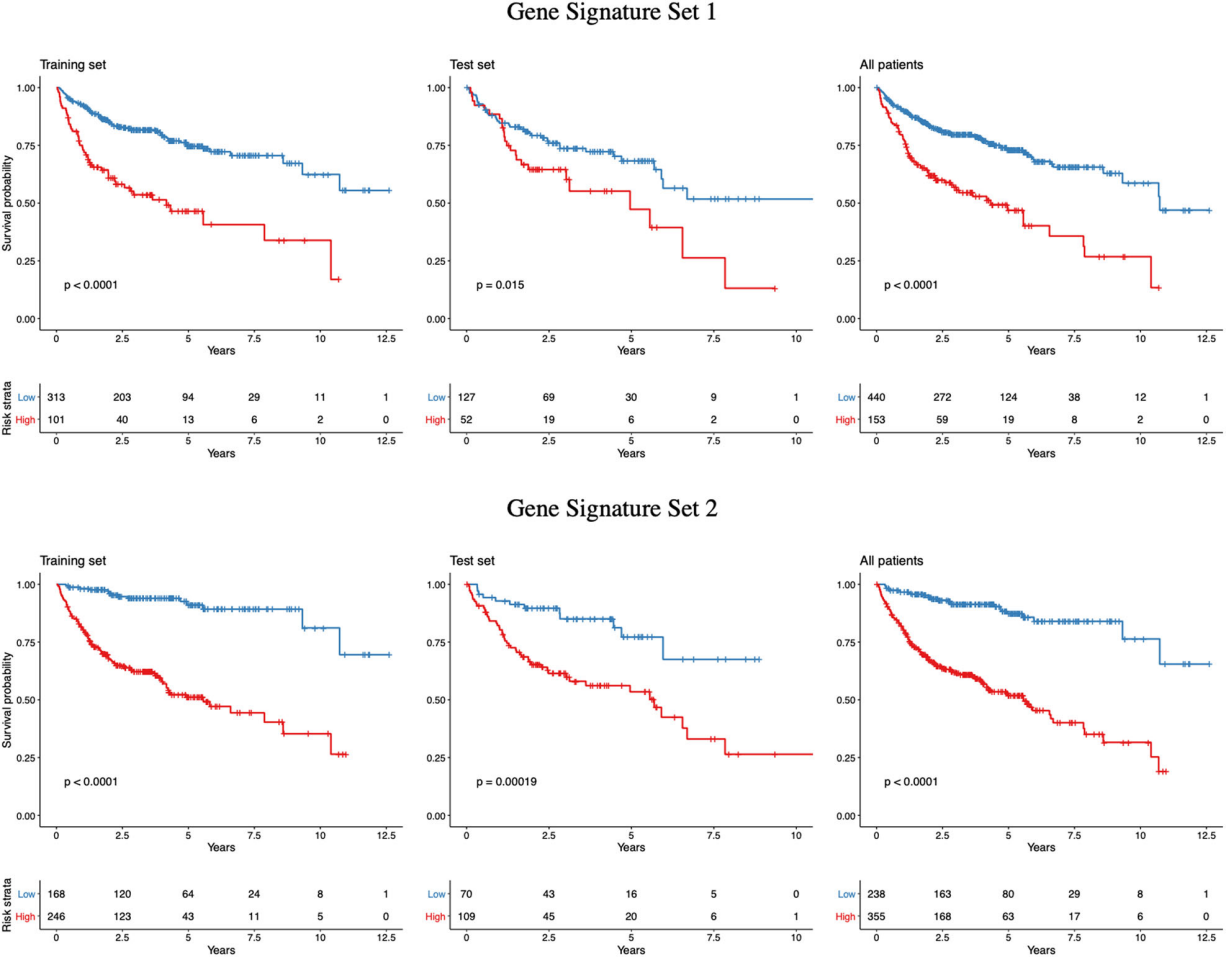
Overall Survival Analysis of Gene Signature Sets 1 and 2. The overall survival among the patients in the training, test, and entire sets according to the optimal cut-offs for the gene expression-based survival-predictor scores obtained from the models with signature sets 1 and 2.

Based on the Lasso feature selection method, four signatures comprised signature set 1 (Favorable 3 and 4, Unfavorable 1 and 2) and signature set 2 (Favorable 1 and 2, Unfavorable 1 and 2). Multivariable Cox models were developed with signature sets 1 and 2 on the training set. The patients in the training set were recruited into the high- and low-risk groups according to the optimal cut-off for survival-predictor scores (0.41 and −0.20 for signature sets 1 and 2 models, respectively). As depicted in Fig. 3, patients with high score showed significantly worse overall survival than those with low score (*p* < 0.0001). The AUC of the 2-, 5-, and 10-year ROC curve achieved 0.69, 0.68, and 0.65 for signature set 1, and 0.78, 0.78, and 0.80 for signature set 2, respectively. The AUC of the ROC curve in the entire set at time points of 2, 5, and 10 years were 0.68, 0.68, and 0.68 for signature set 1 and 0.75, 0.75, and 0.78 for signature set 2, respectively.

Kaplan-Meier plots of overall survival (Fig. 3) showed distinct differences among the risk groups in the test and entire datasets for survival (*p* < 0.02). The survival-predictor scores from these models were highly predictive of survival in validation sets (*p* < 0.001). Moreover, the survival-predictor scores from signature set 2 model resulted in a larger log-likelihood in the validation set, reflecting a higher degree of association with survival (*p* < 0.001). Therefore, signature set 2 was used for further predictive modeling analyses. Each unit increase in the gene expression-based predictor score was associated with an increase in the relative risk of death by a factor of 2.04 (95% CI: 1.49−2.78) in the validation set and by a factor of 2.45 (95% CI: 2.10−2.95) in the entire set.

**Fig. 4:**
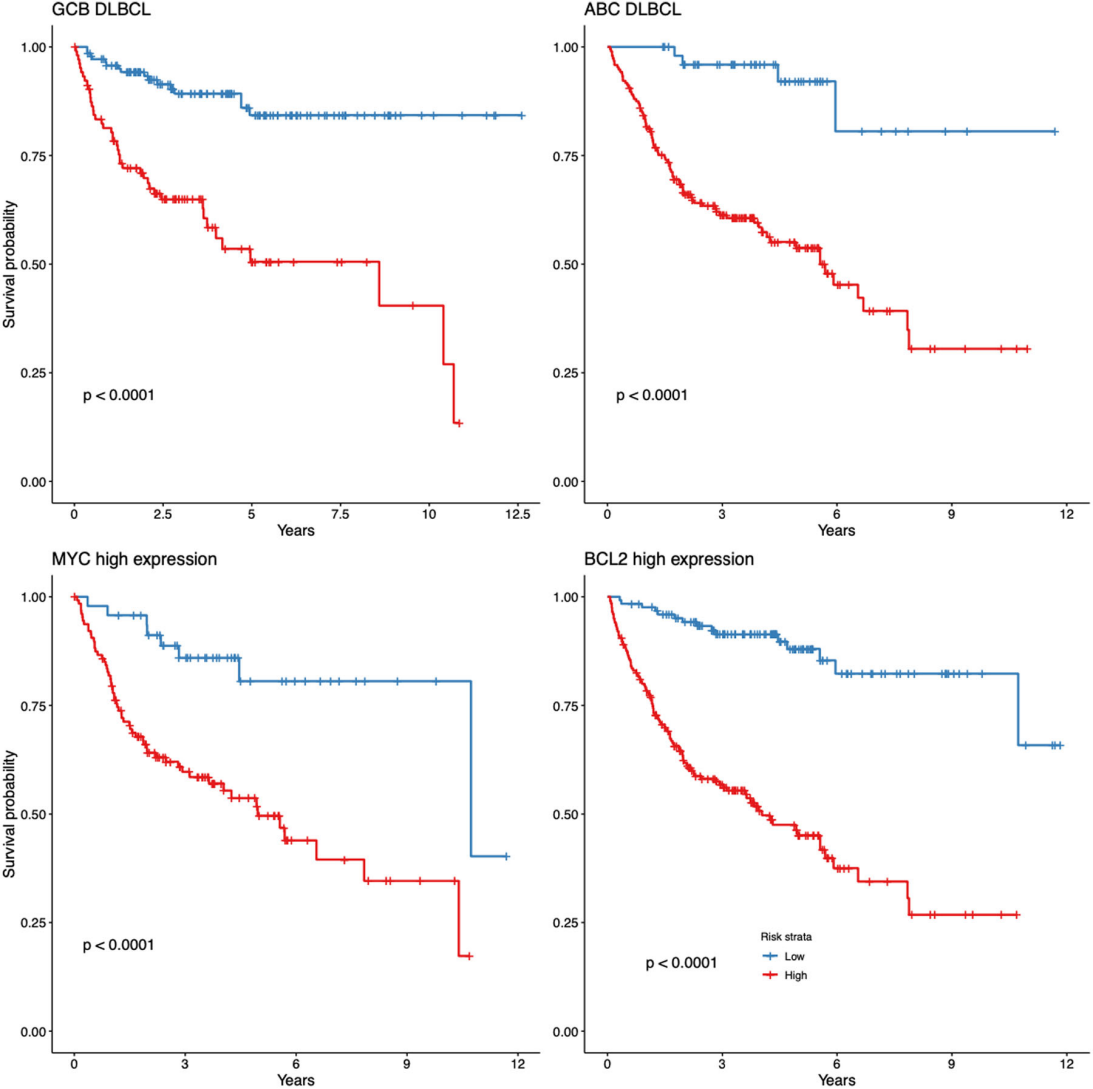
Gene Expression-Based Survival Model. The gene expression-based survival model significantly stratifies survival within ABC and GCB subtypes, and MYC and BCL2 high-expression groups (log-rank test).

The performance of the gene expression-based score was examined in the known clinical risk groups defined by the gene-expression markers (cell of origin, MYC, and BCL2 expression). The gene expression-based method was able to distinguish patients with significantly distinct outcomes across subsets, demonstrating the survival-predictor score’s greater prognostic power as compared with that derived from the use of clinical subgroups of DLBCL (Fig. 4).

### Comparison of the gene expression-based survival predictor and the IPI

**Fig. 5:**
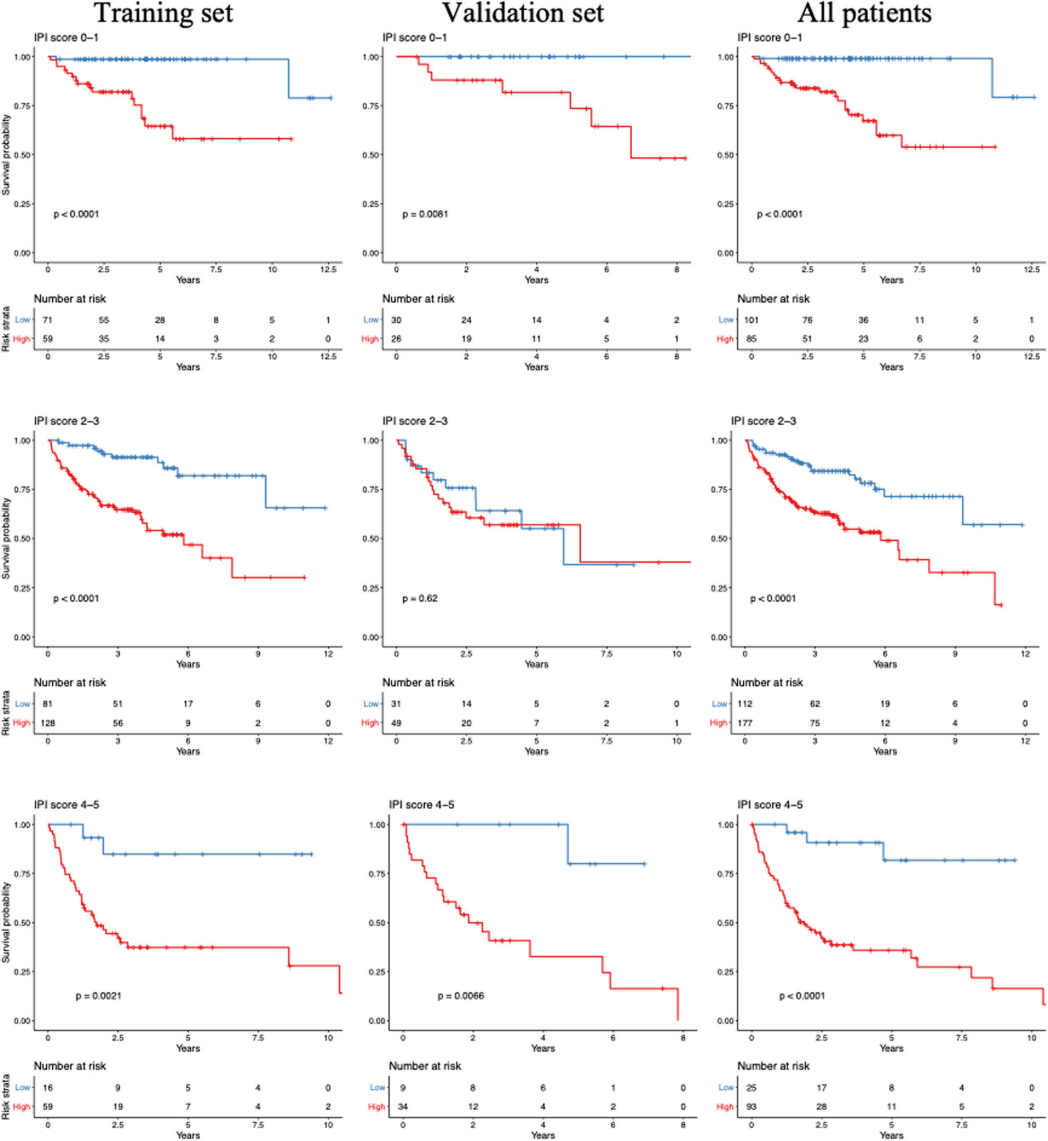
Overall Survival Stratified by IPI Risk Groups. The overall survival among patients in the various IPI risk groups in the training, test, and entire datasets, stratified according to the optimal cut-off for the gene expression-based survival-predictor score.

There were 593 patients with IPI scores available in the core set: 414 patients in the training and 179 patients in the validation sets. In the multivariable Cox models that combined both the IPI scores and the gene expression-based scores from signature set 2, the gene expression-based score was an independent predictor for overall survival in the validation sets (Table 1). Each unit increase in the gene expression-based score increased the relative risk of death on the entire dataset by a factor of 2.23 (95% CI: 1.88−2.66). Kaplan-Meier plots of overall survival showed the independence of the IPI score and the gene expression-based predictor score (Fig. 5).

**Table 1:** Multivariate Cox regression analysis with the gene expression-based predictor score and the IPI for the overall survival of DLBCL patients.

	Training set (*n* = 414)			Test set (*n* = 179)			Entire set (*n* = 593)		
Variables	HR	95% CI	*p*-value	HR	95% CI	*p*-value	HR	95% CI	*p*-value
IPI									
Low	0.31	0.17−0.55	6.92 × 10^−5^	0.2	0.085−0.47	0.0002	0.26	0.16−0.42	3.3 × 10^−8^
Intermediate	0.64	0.42−0.96	0.032	0.69	0.40−1.19	0.18	0.64	0.46−0.88	0.007
High	1	(reference)		1	(reference)		1	(reference)	
Survival-predictor score	2.41	1.96−2.97	*<*2 × 10−16	1.86	1.33−2.56	0.00025	2.23	1.88−2.66	*<* 2 × 10−16

*Abbreviations*: *CI* confidence interval, *HR* hazard ratio.

To evaluate the impact of integrating the gene expression-based predictor score and the IPI score on the prognostic accuracy, time-dependent ROC analysis was conducted using the survival-predictor scores based on the multivariable model developed on the training set with these two predictors. The AUC of the ROC curve on the entire set at time points of 2, 5, and 10 years were 0.79, 0.78, and 0.83, respectively, indicating that the combination of the gene expression-based predictor score with the IPI score improved discrimination on the entire set over the gene expression-based predictor score alone. Moreover, the survival-predictor scores from the model with the gene expression-based predictor score combined with the IPI score resulted in a larger log-likelihood in the validation set, reflecting a higher degree of association with the survival (*p* < 0.001). Based on these findings, the IPI was included together with the gene expression-based predictor score in our final model.

### Biological implications of survival gene signatures

Systems biology analysis revealed endocytosis, focal adhesion, cytokine–cytokine receptor interaction, and MAPK signaling pathway as the major enriched biological pathways for the favorable signatures (Table S6). Similarly, the major enriched biological pathways for the unfavorable signatures were metabolic pathways, spliceosome, RNA transport, and aminoacyl-tRNA biosynthesis. KEGG pathway-enrichment analyses demonstrated that Favorable signature 1 was remarkable for overlap with T-cell dysregulation, particularly CD4 + T-cells, as seen with the CIBERSORT analysis (Table S6). Unfavorable signatures demonstrated findings related to RNA transport and metabolic pathways.

**Fig. 6:**
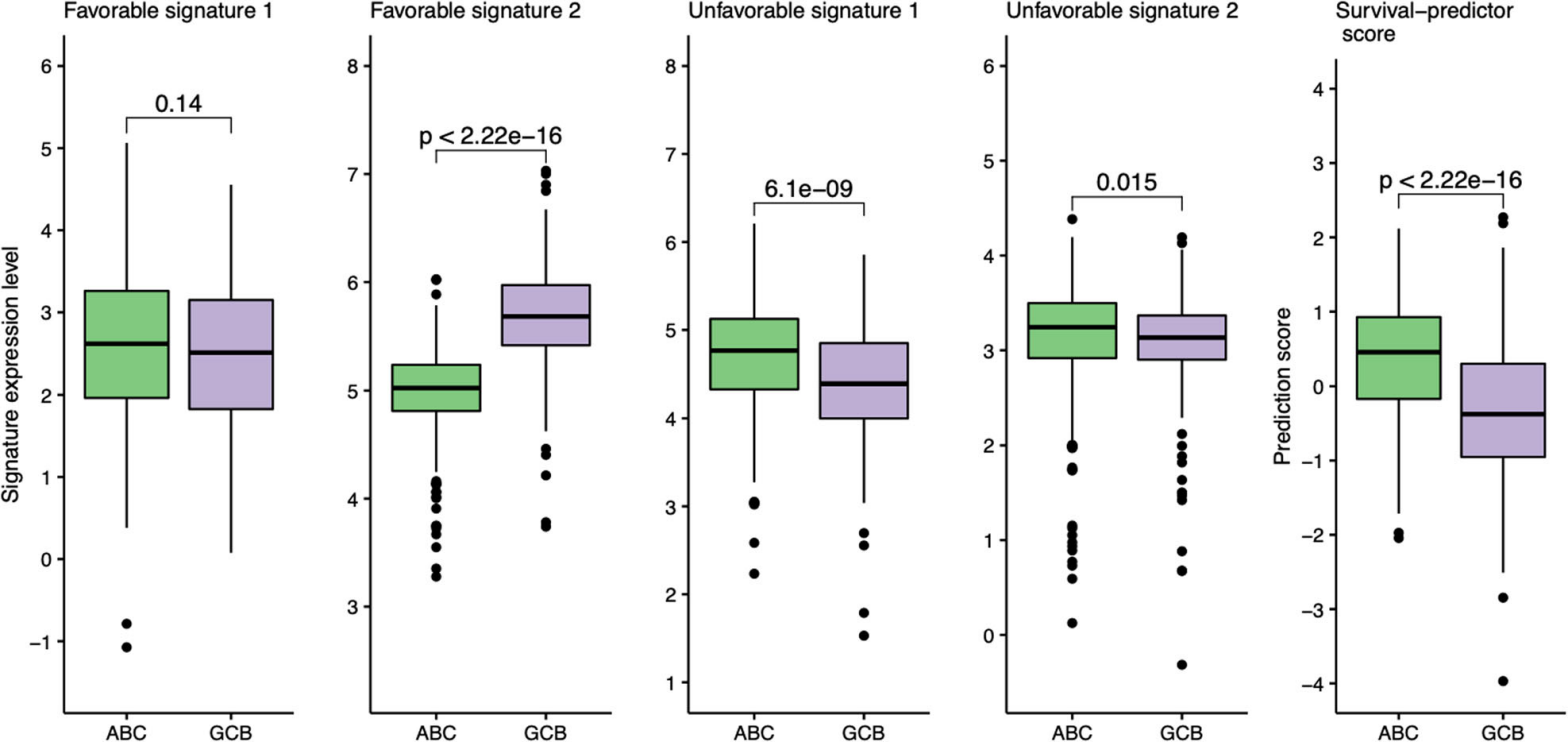
Gene Expression Levels of the Survival-prediction model. The level of expression of gene signatures in the survival-prediction model and the predictor scores in ABC and GCB.

To get extract biological insights into the connection between the gene signatures and cell-of-origin, we investigated whether the components of the survival predictor were differentially expressed between ABC and GCB (Fig. 6). The Favorable signature 2 was more commonly found in GCB than in ABC. The Unfavorable signature 1 was more common in ABC than in GCB. The level of expression of the Favorable signature 1 was similar among these subgroups. The gene expression-based score was higher in ABC than in GCB, supporting our earlier finding that the predictor score could be used to subdivide DLBCL patients in ABC and GCB into distinct risk groups.

Genes in the unfavorable signatures tended to activate the acute immune system response and angiogenesis. Genes in the favorable signatures were enriched for hematopoiesis, and activate Wnt signaling while downregulating adaptive immune response systems (Tables S7–S9).

### Prognostic implications of immune composition

Table S10 and Figs. S6 and S7 present the correlation between the immune cell subtypes and gene signatures identified by the Dynamic Cut Tree method that had the most significant correlation. Resting CD4+ memory T-cells, regulatory T-cells, and M0 macrophages were positively correlated with the favorable signatures. The correlation between the immune cell content and the risk score revealed similar patterns (Fig. S8). KEGG pathway-enrichment analyses demonstrated that activated dendritic cells, neutrophils, eosinophils, mast cells, monocytes, and M1/2 macrophages were correlated inversely with the favorable signatures or positively with the unfavorable signatures (all *p* < 0.0001).

## Discussion

In this study, supervised and unsupervised machine learning methods were implemented to identify gene-expression signatures that were associated with survival and subsequently used to construct a multivariable regression model for predicting the overall survival of DLBCL patients. The gene expression-based predictor scores and the IPI scores were both independent prognostic indicators, and the combination of the two scores improved the identification of high-risk DLBCL patients.

In the risk prediction model, Favorable signature 2 and Unfavorable signature 1 were independent predictors for overall survival (Table 2). The deconvolution analysis of Favorable signature 2 showed a negative correlation of resting NK cells with the genetic signature while the systems biology analysis showed association with stress-activated MAPK signaling cascade and phospholipid translocation. The genes in Unfavorable signature 1 were significantly enriched in pathways associated with IgG binding, neutrophil activation, degranulation, and cell-mediated immunity, supporting an inflammatory malignancy like DLBCL^33^. The negative correlation of this signature with CD4+ naive T-cells, follicular helper T-cells, and activated NK cells may imply the need for successful therapeutic interventions to activate these cell subtypes.

**Table 2:** Multivariate Cox regression analysis with survival gene signatures for the overall survival of DLBCL patients.

		Training set (*n* = 414)			Test set (*n* = 179)			Entire set (*n* = 593)		
Gene-expression variables	No. of genes	HR	95% CI	*p*-value	HR	95% CI	*p*-value	HR	95% CI	*p*-value
Favorable signature 1	86	0.66	0.53−0.81	*<*0.001	0.78	0.58−1.05	0.097	0.68	0.57−0.80	*<*0.001
Favorable signature 2	82	0.49	0.35−0.68	*<*0.001	0.62	0.36−1.06	0.078	0.53	0.40−0.70	*<*0.001
Unfavorable signature 1	92	1.83	1.37−2.43	*<*0.001	1.5	0.95−2.37	0.08	1.73	1.36−2.20	*<*0.001
Unfavorable signature 2	72	2.38	1.64–3.45	*<*0.001	2.26	1.32−3.86	0.003	2.26	1.68−3.03	*<*0.001

*Abbreviations*: *CI* confidence interval, *HR* hazard ratio.

When the deconvolution findings were correlated with the gene signatures and the risk score, three subsets of cells stood out: CD8 + T-cells, naive CD4 + T-cells, and activated dendritic cells. The former two conveyed a favorable prognosis while the latter was associated with poorer prognosis. To explain this pattern of which cells are preferable, cell signaling and interleukin activity were looked into further and the following was inferred as a possible explanation. In non-pathological environments, antigen presenting cells tend to produce IL-12, a pro-inflammatory cytokine with anti-tumor properties that binds to the IL-12R heterodimeric receptor consisting of IL-12R*β*1 and IL-12R*β*2^34^. Both subunits of the receptor are expressed in activated T-cells and NK cells but only IL-12R*β*1 is expressed on naive T-cells^34^. When the IL-12R*β*1 subunit is combined with the Ebi3 subunit, it becomes an immunosuppressive unit activated by IL35^35^. IL-35, a pro-tumor member of the IL12 cytokine family, is overexpressed in DLBCL^36^ and suppresses naive T-cell activation^37^. In a phase II clinical trial for non-Hodgkin’s Lymphoma and Hodgkin’s Lymphoma, IL12 administration increased circulating CD8+ T-cell presence but had no effect on CD4+ T-cell presence^38^. Alternately, IL35 suppression will likely increase T-cell activation and presence.

CD8 + T-cells are usually suppressed in the TME through an enhanced TGF-*β* pathway^39^, which suppresses the immune response and enhances inflammatory signals and carcinogenesis^40^. We found that TGFB1I1 gene, which codes for the first subunit of the TGF-*β*1 protein, was part of the good prognosis signature 2 gene set and warranted further attention. Li and Flavell^41^ published a three-cell model for T-cell regulation from the TGF-*β*1 pathway. Per this model, Tregs secrete latent TGF-*β*1 after activation by antigen presenting dendritic cells. The latent protein is processed by av8 integrins on the cell membranes of dendritic cells into the active TGF-*β*1 form, which inhibits the differentiation of naive CD4+ T-cells into Th1 or Th2 cells. Instead, TGF-*β*1 promotes the differentiation of naive CD4+ T-cells into regulatory T-cells and Th17 cells through a SMAD1-dependent pathway^41^. TGF-*β*1 inhibits the production of IL-12^42^. In DLBCL, TGF-*β* pathway is inactivated at the level of SMAD1^43,44^ such that downstream enhancement of this pathway would likely benefit survival outcomes.

Genes in the unfavorable signatures tended to activate the acute immune system response and angiogenesis, both of which are associated with metastatic disease and poor prognosis through a constitutively activated STAT3 pathway^45^. Genes in the favorable signatures tended to promote organ development and hematopoiesis, which may be associated with cell differentiation, and activate Wnt signaling while downregulating adaptive immune response systems. Wnt signaling in conjunction with TGF-pathway is associated with the development of mature hematopoietic stem cells^46^, which has therapeutic implications in hematological malignancies such as DLBCL. For instance, harvesting stem cells from healthy bone marrow donors and transplanting them into the patient allows for new healthy development of all blood cell lines which were likely depleted^46^. Correlating these signaling processes with the deconvolution analysis assists in putting perspective to the findings.

A number of genetic signatures and prognostic models have been published in the last few years^2,3,9,47,48^. Prognostic algorithms which are genetically focused on the malignant cells and do not incorporate the TME in their validation are at risk of being inaccurate and misleading because signaling between the tumor and its microenvironment can affect the nature and progression of the malignancy^49^. For instance, Hazlehurst et al.^50^ showed TME-induced resistance by fibronectin to cell adhesion-mediated therapeutic intervention in myeloma. The TME and tumor co-evolve in B-cell malignancies, allowing for multiple routes of tumor growth and progression, immune evasion, and cell death resistance^51^. Indeed, modulating the TME can have profound effects and can be exploited therapeutically as in the case of lenalidomide in follicular lymphoma or PD-1 blocking antibodies in Hodgkin Lymphoma^52^.

Our algorithm incorporates IPI scores and is supported by CIBERSORT, something which has not been done by other published models. Ciavarella et al.^53^ attempted to incorporate CIBERSORT into their algorithm which worsened prognostic prediction in the context of formalin-fixed paraffin-embedded tissue samples. Biccler et al.^54^ constructed a prognostic “stacking” model which takes advantage of established prognostic models and builds on them. Similar to our construct, they compared their stacking model with the Cox proportional hazard model with the IPI variables (CPH-IPI) and found that the stacking model was superior when non-IPI clinical factors were included in the algorithm. When considering real-life application of an algorithm, gaining insight on the TME and estimating survival outcomes is more beneficial, which our algorithm provides.

In conclusion, we developed and validated a robust survival-prediction model which may facilitate the prognostic evaluation and risk stratification of patients with DLBCL. Our analysis of immune cell subsets in DLBCL has revealed important associations with the clinical outcomes. Coupling the changes noted in immune cell content of the TME with the reliable risk predictions can aid personalized decision making regarding individual disease course and treatment outcomes.

## Supplementary information

Supplementary Material

## Data Availability

Please email the corresponding authors for access to codes used for the analysis described above.
