# Supplementary material for: Gene expression profiling-based risk prediction and profiles of immune infiltration in diffuse large B-cell lymphoma
Source: Blood Cancer J. 2021 Jan 7;11(1):2. doi: 10.1038/s41408-020-00404-0 (PMC7791044; doi:10.1038/s41408-020-00404-0)
Supplement: Supplementary file 1 — Supplementary Material [file 41408_2020_404_MOESM1_ESM.pdf]

Gene expression profiling-based risk prediction and profiles of immune infiltration in diffuse large B-cell lymphoma: Supplementary Material

Merdan and Subramanian et al.

Characteristics of diffuse large B-cell lymphoma patients

Table S1 summarizes the clinical characteristics of 718 patients with complete overall survival information.

Table S1: Clinical characteristics of the entire patient cohort.

**Variables**	**Number of patients (n = 718)**	**Variables**	**Number of patients (n = 718)**
IPI		CNS involvement	
Low	186 (25.9)	Yes	16 (2.2)
Medium	289 (40.3)	No	629 (87.6)
High	118 (16.4)	Unknown	73 (10.2)
Unknown	125 (17.4)	CNS relapse	
ECOG IPI		Yes	33 (4.6)
Yes	181 (25.2)	No	282 (39.3)
No	496 (69.1)	Unknown	403 (56.1)
Unknown	41 (5.7)	Age	
Ann Arbor stage IPI		Mean (IQR)	60.9 (51.7, 72.3)
Yes	429 (59.7)	≤ 60	
No	279 (38.9)	> 60	
Unknown	10 (1.4)	Overall survival	
LDH IPI		Mean (IQR)	3.5 (1.4, 5.0)
Yes	368 (51.3)	MYC high expression	
No	291 (40.5)	Yes	399 (55.6)
Unknown	59 (8.2)	No	300 (41.8)
Multiple extranodal IPI		Unknown	19 (2.6)
Yes	163 (22.7)	BCL2 high expression	
No	510 (71.0)	Yes	396 (55.2)
Unknown	45 (6.3)	No	322 (44.8)
Testicular involvement		BCL6 high expression	
Female	311 (43.3)	Yes	374 (52.1)
Yes	31 (4.3)	No	344 (47.9)
No	300 (41.8)	Cell of origin	
Unknown	76 (10.6)	ABC	295 (41.1)
B symptoms at diagnosis		GCB	301 (41.9)
Yes	244 (34.0)	Unclassified	122 (17.0)
No	440 (61.3)	Response to initial therapy	
Unknown	34 (4.7)	Complete response	556 (77.4)
		Partial response	62 (8.6)
		No response	58 (8.1)

Abbreviations: IPI, International Prognostic Index; ECOG: Eastern Cooperative Oncology Group; LDH, Lactate dehydrogenase; CNS, central nervous system; GCB, germinal center B-cell; ABC, activated B-cell; IQR: inter quartile range.

Gene expression profiling

Hierarchical clustering was applied to gene expression data using the unweighted pair-group method as implemented in the R `hclust` package. The distance matrices used were Pearson correlation for clustering the arrays. We examined the cluster sizes to determine a criteria on the clusters to be included in the statistical analyses. The distribution of the number of genes in the pre-defined cutoff clustering method are illustrated in Figure S1. The Dynamic cut tree method identified 15 and 12 clusters of genes that were associated with favorable and poor prognosis, respectively, with the sizes 20, 24, 36, 41, 47, 48, 53, 69, 72, 82, 86, 118, 128, 143, 171, and 22, 28, 30, 44, 48, 61, 69, 70, 83, 92, 148, 154, respectively. These results together with our aim to develop a practical risk prediction tool for clinical use, each gene signature was defined using as a cluster that met the following criteria on cluster size: the presence of at least 20 genes and no more than 100 genes in the cluster. Figures S2 and S3 illustrate the gene expression profiles and hierarchical clustering of the good-prognosis and poor-prognosis genes where the clusters are determined using a fixed dissimilarity cutoff of 0.4. Figures S4 and S5 illustrate the gene expression profiles and hierarchical clustering of the good-prognosis and poor-prognosis genes where the clusters are detected by the Dynamic Cut Tree method [1]. For visualization, gene expression data were centered by subtracting the median observed value.

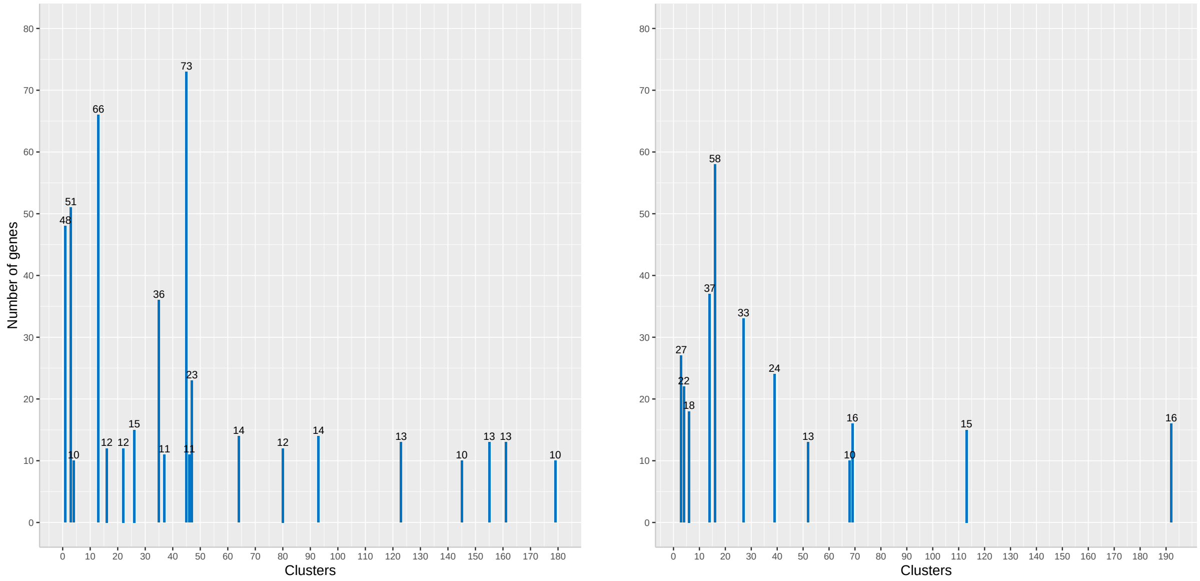

Figure S1: The distribution of cluster sizes identified by the pre-defined cutoff clustering method.

Figure S2: Gene expression profiling of good-prognosis genes and the hierarchical clustering with gene clusters identified at the dissimilarity cutoff of 0.4.

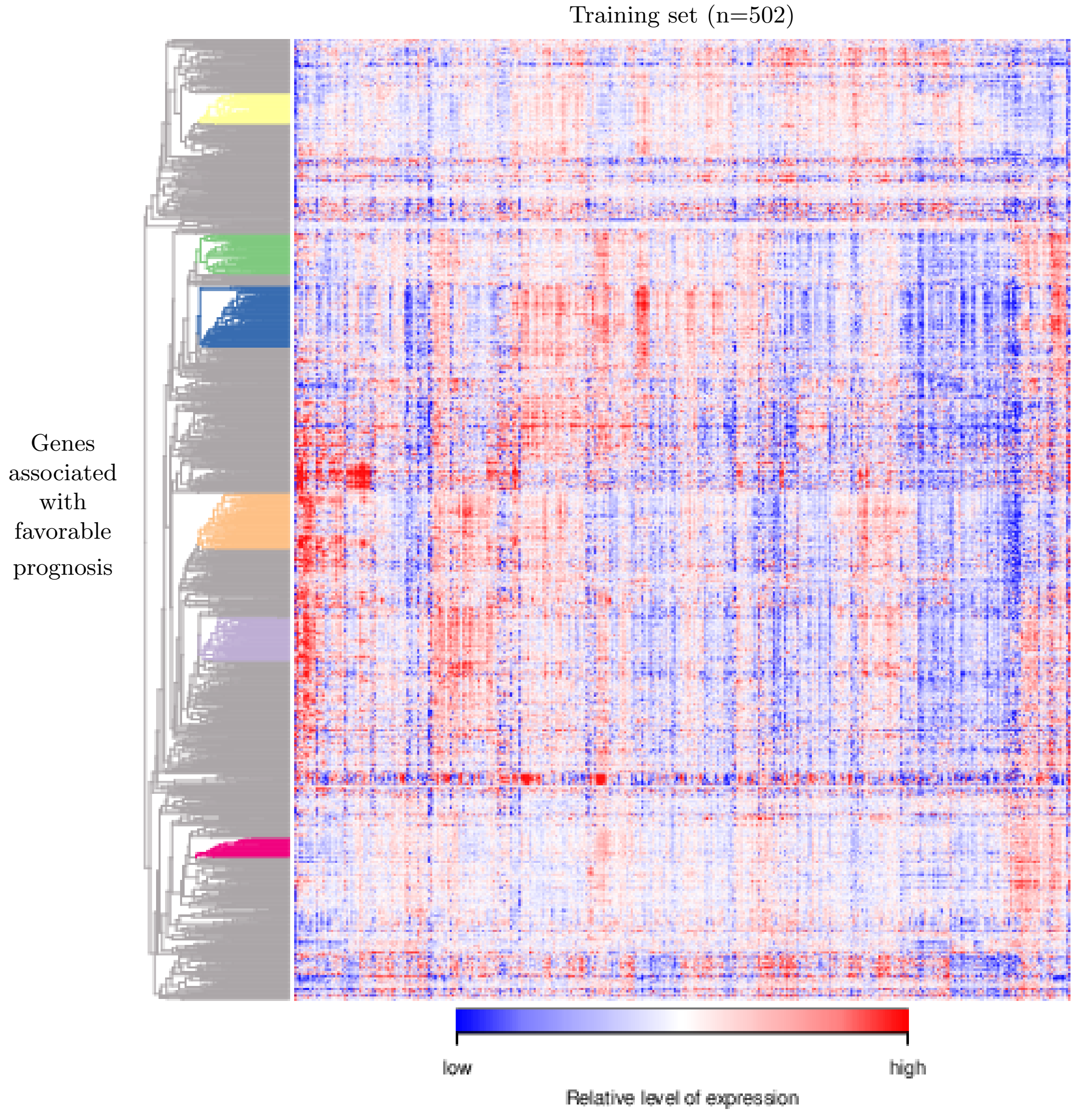

Figure S3: Gene expression profiling of poor-prognosis genes and the hierarchical clustering with gene clusters identified at the dissimilarity cutoff of 0.4.

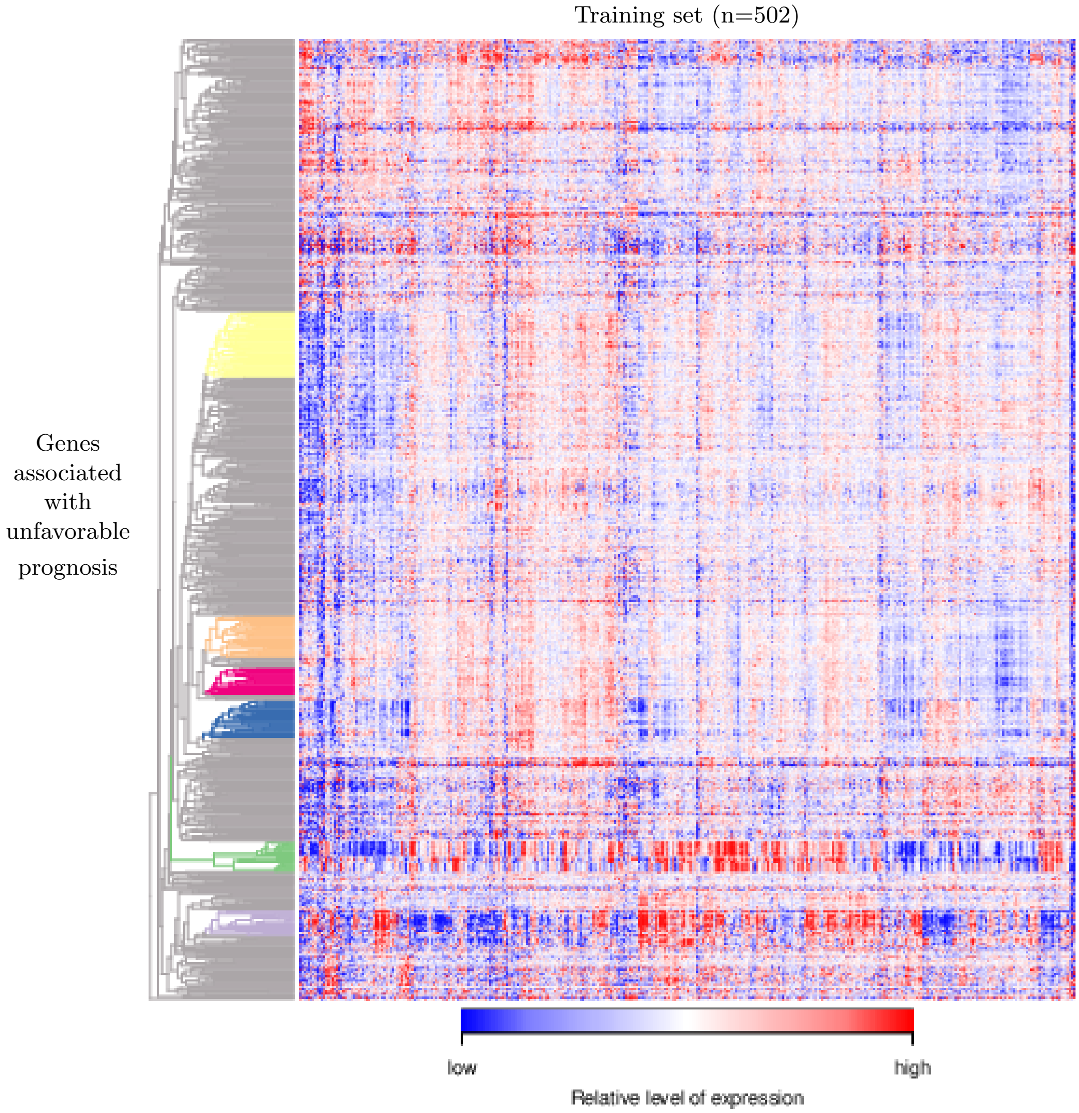

Figure S4: Gene expression profiling of good-prognosis genes and the hierarchical clustering with gene clusters identified by the Dynamic Cut Tree method.

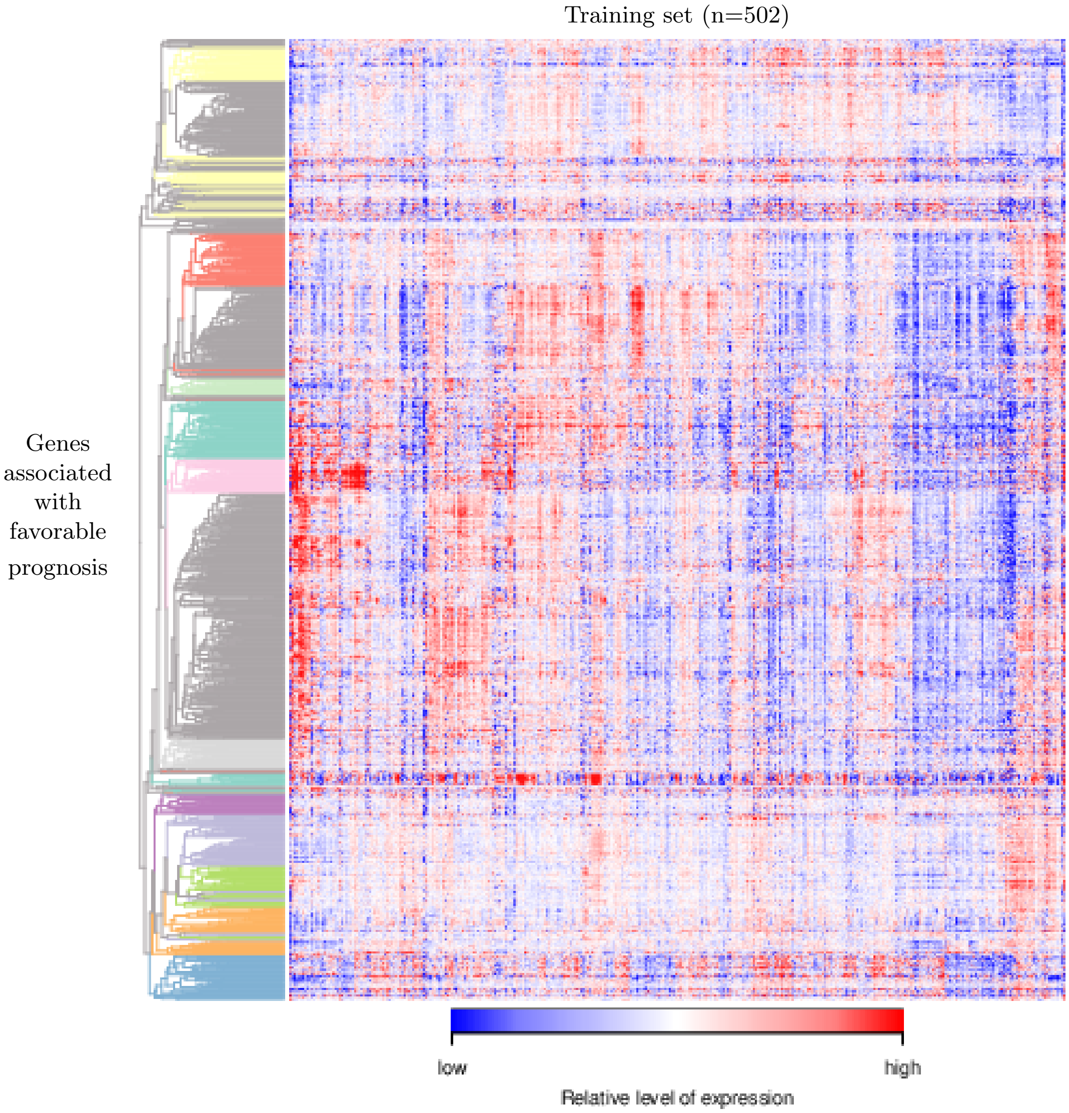

Figure S5: Gene expression profiling of poor-prognosis genes and the hierarchical clustering with gene clusters identified by the Dynamic Cut Tree method.

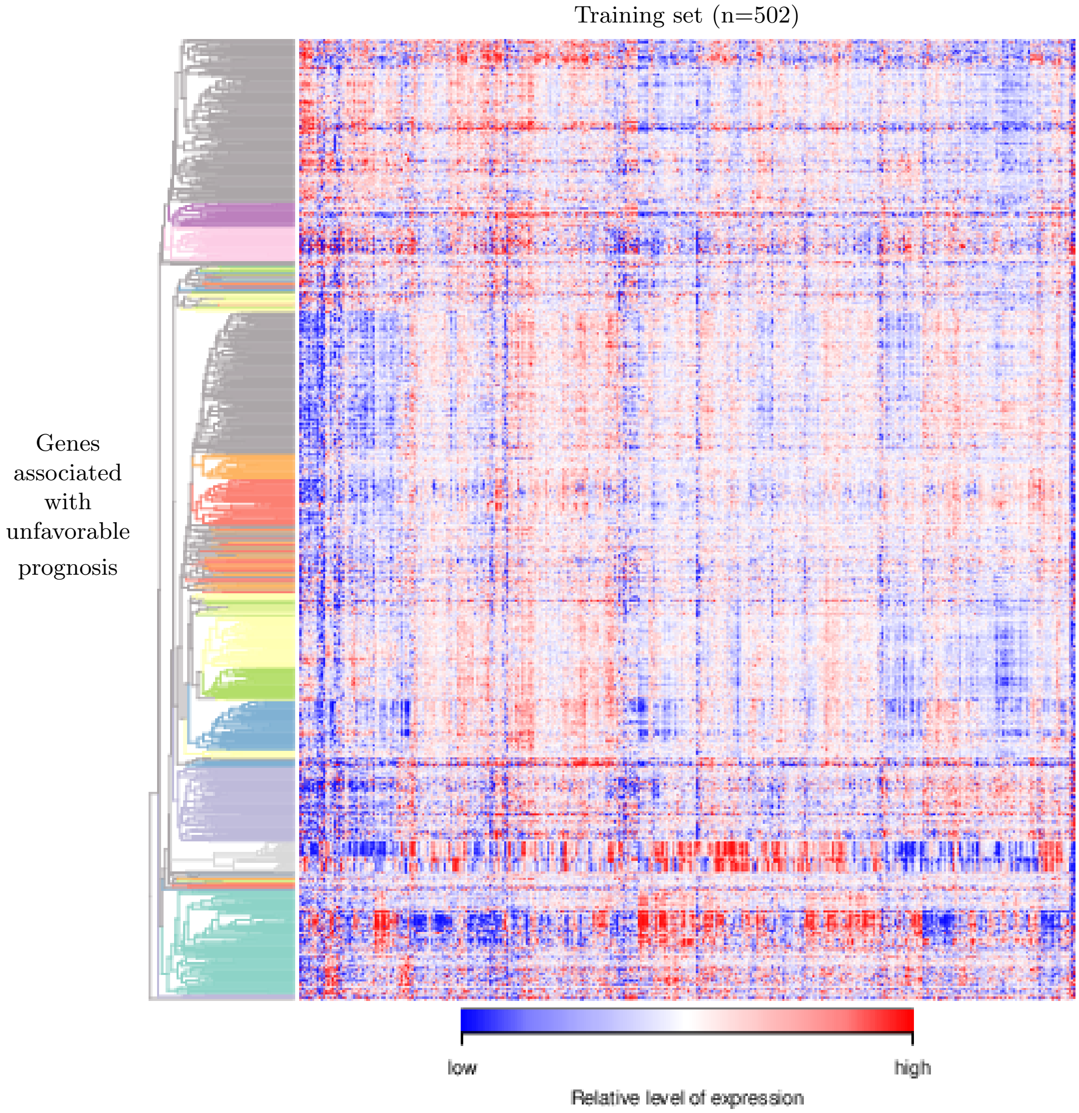

Gene signatures and gene enrichment analysis

Table S2 shows the members of the gene clusters detected at the correlation cutoff of 0.4 and the gene ontology of each cluster.

Table S2: The gene signatures identified at the minimal correlation cutoff and associated with favorable and unfavorable prognosis.

Signature	Member genes	Gene Ontology
	**Good-prognosis gene signatures**	
Signature 1	TNFRSF18, TNFRSF4, PLCH2, TNFRSF25, LDLRAP1, TTC22, CSF1, NOTCH2NLA, VSIR, SIGIRR, LTBP3, BCL9L, CD4, BIN2, BTBD11, SELPLG, GPR68, TNFAIP2, GPR132, PLEKHO2, MAN2C1, HAPLN3, MMP25, IL32, NLRP1, ACAP1, HS3ST3A1, HS3ST3B1, SREBF1, MLLT6, SGSH, SBNO2, JAK3, PLEKHF1, CEBPA, NPTXR, UBA7, ZFYVE28, TIFAB, HLA-F, HLA-E, HLA-B, TAPBP, GIMAP1, FAM160B2, RALGDS, NOTCH1, ABCA2	Cellular response to cytokine stimulus, cytokine-mediated signaling pathway, response to cytokine, antigen processing and presentation of endogenous antigen, integral component of lumenal side of endoplasmic reticulum membrane, peptide antigen binding, Golgi membrane, cellular response to organic substance, defense response, response to organic substance
Signature 2	MXRA8, MEGF6, PLEKHG5, EPHA2, COL16A1, LMNA, NES, DDR2, NAV1, PTPN14, FAM171A1, SVIL, UNC5B, MARVELD1, CACNA1C, GRASP, TNS2, EFS, CRIP2, GLIS2, TGFB1I1, MTSS2, ZNF469, HIC1, ARHGAP23, SPHK1, C1QTNF1, SHC2, ZNF358, SYDE1, EHD2, RAB11FIP5, GLI2, COL18A1, SCARF2, CDC42EP1, PDGFB, FLNB, AMOTL2, AFAP1, TMEM173, FLT4, DST, TPBG, PCOLCE, ZNF703, ADGRA2, ZNF462, FAM129B, NCS1, IQSEC2	Locomotion, regulation of cellular localization, ameboidal-type cell migration, anatomical structure development, regulation of cellular component movement, multicellular organism development, cell motility, cytoskeleton organization, developmental process, regulation of signaling
Signature 3	KAZN, GNG12, PBX1, DPT, PRRX1, JCAD, ADAM12, PARVA, FIBIN, CTNND1, PGR, YAP1, CLMP, ARHGAP32, PPFIBP1, LIMA1, FLRT2, PTPN21, TJP1, EMP2, CDH11, MPP3, PLEKHH2, ANTXR1, NPAS2, IL1R1, SOWAHC, TANC1, PLA2R1, NCKAP1, MYO1B, RIN2, RBMS3, ZNF662, ROBO1, DCBLD2, ABI3BP, MYLK, WWTR1, LGI2, LIMCH1, PDLIM5, SH3D19, GUCY1A1, WWC2, SEMA5A, GPX8, IL6ST, ARHGEF28, PAM, PPIC, DPYSL3, GJA1, PTPRK, ENPP1, EGFR, CALD1, ASPH, CYP7B1, GEM, COL14A1, BNC2, PTPN3, LPAR1, NHS, PLS3	Anatomical structure morphogenesis, actin cytoskeleton, developmental process, plasma membrane, cell projection organization, actin filament organization, actin cytoskeleton organization, cell periphery, actin filament-based process, animal organ morphogenesis
Signature 4	PBXIP1, ARHGAP30, FBXW4, ANO9, CCDC88B, SLC25A45, TBC1D10C, ARHGAP9, NLRC3, RNF166, FMNL1, TMC6, TMC8, DENND1C, RASAL3, ARHGEF1, ZNF831, SUN2, FCHSD1, GRK6, ZMIZ2, NUDT18, AKNA	GTPase activator activity, positive regulation of GTPase activity, GTPase regulator activity, regulation of GTPase activity, ion channel activity, enzyme activator activity, mechanosensitive ion channel activity, channel activity, passive transmembrane transporter activity, regulation of small GTPase mediated signal transduction
	**Poor-prognosis gene signatures**	
Signature 1	C1QA, C1QC, C1QB, FCGR1B, FCGR1A, FCGR2A, FCGR3A, FCGR2C, FCGR3B, FCGR2B, MS4A4A, CASP5, CD163, AQP9, MT1G, PSTPIP2, LILRA6, MARCO, SMIM25, MSR1, ACO1, VSIG4	IgG binding, immunoglobulin binding, immune response, endocytosis, receptor-mediated endocytosis, activation of immune response, phagocytosis, collagen trimer, immune system process, innate immune response
Signature 2	TTC4, COPA, POLR3A, MRPL17, POLD3, NDUFA9, DDX47, PA2G4, CNPY2, PWP1, MRPL57, NOMO1, NOMO3, NOMO2, OGFOD1, CNOT1, ATP6V0D1, UTP4, SF3B3, USP10, TUBG1, NOL11, EIF4A3, PSMD8, PSMC4, CEBPZ, NOP58, FARSB, RPN2, GART, SEC61A1, COPG1, SRPRB, MRPS18A, GARS, TSTA3, PSMB7	rRNA metabolic process, ribosome biogenesis, rRNA processing, ribonucleoprotein complex biogenesis, protein-containing complex, amide biosynthetic process, intracellular membrane-bounded organelle, cellular amide metabolic process, proteasome complex, catalytic complex
Signature 3	OMA1, INTS7, NVL, NUDT5, MTPAP, ECD, SEC23IP, ABRAXAS2, MTCH2, NUP160, LOC728715, TMEM106C, SENP1, XPOT, FAM216A, EIF2B1, UCHL3, VRK1, HACD3, VPS35, RSPRY1, NAE1, TMEM97, MRPL45, ME2, NARS, ZNF121, ZNF724, ZNF543, ADI1, SF3B6, COMMD1, MRPL30, OLA1, ATP5MC3, WDR75, PMS1, ACSL3, CSNK2A1, CSE1L, GABPA, SPICE1, QTRT2, NMD3, ZGRF1, IPO11, GFM2, EEF1E1, PPIL1, BAG2, RPF2, FBXO5, MTERF3, NUDCD1, MRPL13, PUM3, IARS, INIP	Intracellular membrane-bounded organelle, membrane-bounded organelle, mitochondrion, intracellular organelle, cellular nitrogen compound metabolic process, protein localization to nucleolus, nucleus, nitrogen compound metabolic process, cellular metabolic process, mitochondrial translation
Signature 4	CHD1L, KIF14, KIF18A, FOXM1, TROAP, TIMELESS, NUP107, KNTC1, DIAPH3, DLGAP5, KIF23, TICRR, TEDC2, PALB2, RFWD3, CDC6, TOP2A, BRCA1, BIRC5, ANKLE1, CCNE1, MSH2, ANAPC1P1, ANAPC1, POLR1B, CCDC150, MCM8, AURKA, DONSON, POLQ, TOPBP1, NUP155, KIF24	Cell cycle phase transition, mitotic cell cycle, mitotic cell cycle phase transition, cell cycle, mitotic cell cycle checkpoint, regulation of mitotic cell cycle, regulation of cell cycle process, DNA metabolic process, regulation of cell cycle, cellular response to DNA damage stimulus
Signature 5	CCT3, EPRS, SEPHS1, CKAP5, PRPF19, ATP5F1B, SHMT2, IPO5, NDUFAB1, NUP93, CSNK2A2, BLMH, PHB, DDX1, LRPPRC, RUVBL1, HADH, HSPA9, GEMIN5, CDC5L, TCP1, BZW2, POLD2, PTDSS1	Nucleoid, mitochondrial nucleoid, myelin sheath, mitochondrial matrix, nuclear lumen, carboxylic acid catabolic process, small molecule binding, chromosome, ribonucleoprotein complex, organic cyclic compound binding

Table S3 and S4 show the member genes of the gene clusters detected by the Dynamic Cut Tree method and the gene ontology of each cluster.

Table S3: Eight gene signatures identified by the Dynamic Cut Tree and associated with favorable prognosis.

**Signature**	**Member genes**	**Gene Ontology**
Signature 1	CADM3, VANGL2, LAMC2, NFASC, RIC3, SLC1A2, ARHGAP20, NCAM1, PKNOX2, IGSF9B, CLEC9A, ETFBKMT, GRIP1, NDRG2, NOVA1, GALNT16, NRXN3, IGHG4, IGHG2, IGHG1, IGHG3, FAM30A, IGHV3-7, IGHV3-21, IGHV3-30, IGHV3-33, IGHV4-59, IGHV1-69, GCOM1, ABCA3, NKD1, FOXF1, MEOX1, TOM1L1, HLF, HSF5, MAP2K6, CDC42EP4, SDK2, ANKRD29, SETBP1, ALPK2, FBXO27, TRABD2A, IGKV1-5, FBLN7, SCN3A, SCN9A, SNPH, C2CD2, IGLV2-14, IGLV3-1, SUSD5, MAATS1, ADCY5, LAMP3, FDCSP, ADAMTS3, MMRN1, CCSER1, LEF1, ANK2, RNF150, NR2F1, KIAA0825, LIX1, CXCL14, WIPF3, PCLO, SEMA3A, RELN, EPHX2, CLU, SCARA3, TNFRSF11B, CCL19, CCL21, PGM5, MUSK, OLFM1, RAI2, ADGRG2, PCDH11X, COL4A5, PAK3, FGF13	Extracellular space, complement activation, classical pathway, complement activation, immunoglobulin complex, circulating, immunoglobulin receptor binding, regulation of complement activation, humoral immune response mediated by circulating immunoglobulin, regulation of protein processing, regulation of acute inflammatory response, regulation of humoral immune response
Signature 2	ZBTB8OS, SYT11, SLAMF1, FAM129A, RGS13, BPNT1, GPR137B, PIP4K2A, CREM, ENO4, RRAS2, PDGFD, DDI1, PCED1B.AS1, ANKRD13A, KLF12, DOCK9, NRL, ZFP36L1, FOXN3-AS1, KLC1, GCHFR, MYEF2, TMOD2, TEX9, TERF2IP, IRF8, TMEM256, FAM106CP, NBR2, CIRBP, LPIN1, FAM49A, NCOA1, MAP4K4, CACNB4, COBLL1, WIPF1, EPHA4, COL4A4, COL4A3, MTERF4, DDX17, C22orf46, VGLL4, GRAMD1C, TIGIT, ZBTB38, MED12L, IGSF10, KLHL6, ATP8A1, ATP10D, HOPX, MAPK10, OTULIN, ANKH, DNAH8, FAM184A, SNX9, HDAC9, PIK3CG, FAM3C, CNTNAP2, TNKS, LONRF1, PPP3CC, DUSP4, RAB11FIP1, ASH2L, TOX, MYBL1, SLCO5A1, PAG1, SAMD12, SLC1A1, TBL1X, SH3KBP1, PDK3, NUDT10, FAM104B, NUP62CL	Stress-activated MAPK cascade, stress-activated protein kinase signaling cascade, negative regulation of telomere maintenance, glomerular basement membrane development, phospholipid translocation, phospholipid-translocating ATPase activity, phosphatidylinositol phosphate kinase activity, ephrin receptor binding, positive regulation of transcription by RNA polymerase II, positive regulation of cellular, metabolic process
Signature 3	TNFRSF8, DDI2, TRIM62, ZC3H12A, SLC39A1, PBXIP1, ARHGAP30, FBXW4, NFKB2, ITPRIP, ANO9, NAP1L4, DDB2, CCDC88B, SLC25A45, TBC1D10C, CORO1B, VAMP1, FMNL3, ARHGAP9, RABGGTA, ST20.AS1, WASH3P, NLRC3, SOCS1, RNF166, SLC16A13, TMEM102, CHD3, EPOP, PGAP3, FMNL1, SP2, TSPOAP1.AS1, TMC6, TMC8, DENND1C, CDKN2D, RASAL3, PBX4, IGFLR1, RINL, RAB4B, EGLN2, ARHGEF1, SPHK2, AKT1S1, TBC1D17, IL4I1, ANKRD23, PTPN18, UBOX5, ZNF831, PISD, SUN2, TTLL3, CCDC12, NBEAL2, GLYCTK, DGKQ, ALPK1, EXOC3, FCHSD1, GRK6, TTBK1, ZC3H12D, ZMIZ2, NUDT18, PDLIM2, TRIM14, AKNA, TRAF1	GTPase activator activity, GTPase regulator activity, positive regulation of GTPase activity, cytosol, retrograde transport, endosome to Golgi, enzyme activator activity, cytoplasm, regulation of GTPase activity, tertiary granule membrane, enzyme regulator activity
Signature 4	TNFRSF18, TNFRSF4, PLCH2, TNFRSF25, GPR157, LDLRAP1, LCK, TTC22, CSF1, NOTCH2NLA, CR1L, PSEN2, VSIR, SIGIRR, LTBP3, BCL9L, CD4, BIN2, BTBD11, SELPLG, RASA3, GPR68, EVL, TNFAIP2, GPR132, PLEKHO2, MAN2C1, HAPLN3, MMP25, IL32, ITGAL, ADCY7, NLRP1, ACAP1, PLSCR3, PIK3R6, HS3ST3A1, HS3ST3B1, SREBF1, MLLT6, PITPNC1, SGSH, B3GNTL1, SBNO2, MATK, JAK3, PLEKHF1, CEBPA, CYP26B1, CSF2RB, NPTXR, MEI1, UBA7, SLC49A3, ZFYVE28, IL7R, TIFAB, HLA-F, HLA-E, HLA-B, TAPBP, LOC154761, GIMAP1, FAM160B2, RALGDS, NOTCH1, ABCA2, FUT7, MECP2	Leukocyte differentiation, T cell activation, signal transduction, cellular response to cytokine stimulus, T cell differentiation, regulation of leukocyte mediated cytotoxicity, intrinsic component of membrane, response to stimulus, response to cytokine, regulation of adaptive immune response
Signature 5	FCRL3, FCRL1, CR2, C1orf115, MARC2, ITPKB, ASB13, LMO2, MS4A1, PACS1, GDPD5, HTR3A, PCED1B, USP12, RASL11A, TTC9, PLD4, CCDC102A, ADGRG5, EFCAB5, RAB11FIP4, SSTR2, MOB3A, S1PR2, LYL1, SLC25A42, CD22, KIF3B, HUNK, LINC01547, ADARB1, MRTFA, SHISA8, TNFRSF13C, RFTN1, CRTAP, CACNA2D3, LSAMP, HEG1, SLC12A8, BFSP2, UCHL1, HLA-DOB, CPNE5, TIAM2, CLIP2, PXDNL, TSPYL5, DENND3, NEK6, FAM102A, XKRX, FRMPD3	B cell proliferation, B cell activation, axo-dendritic transport, axonal transport, axon cytoplasm, regulation of B cell proliferation, negative regulation of immunoglobulin production, regulation of B cell receptor signaling pathway, negative regulation of B cell receptor signaling pathway, neuronal cell body
Signature 6	CRYBG2, PATJ, LAD1, PLEKHA6, AFAP1L2, FGFR2, MICALCL, EHF, RARG, GJB2, TGM1, DIO2, AHNAK2, CDH3, SOX15, TRIM16L, RAPGEFL1, TNS4, RNF43, KCTD1, DSC2, VSIG10L, FAM84A, MAL, ARHGEF4, PTK6, MST1R, CSTA, EPHB3, CLDN1, HPGD, FAT2, DSP, ELOVL4, MAP7, PERP, HR, MOB3B, AQP3, CLIC3, SHROOM2	Epithelial cell differentiation, skin development, keratinocyte differentiation, keratinization, epidermal cell differentiation, cell-cell junction, epidermis development, epithelium development, cornification, cell-cell junction organization
Signature 7	ARHGEF19, IBA57, GNG4, PYROXD2, WNT10B, GAS6, NPAS3, GOLGA8T, GOLGA8H, CCDC9B, MAPKBP1, SPTBN5, FBXL16, LMF1, VASN, SLC52A1, MYO15A, HEXIM1, SAMD14, PCP2, PNMA8B, KLHL29, GPR35, TBX1, POM121L10P, PLCD1, ACKR2, NKD2, ADGRB1, IL11RA, POMT1, ADAMTSL2, DBH, SARDH, SLC34A3, PDZD4	Golgi organization, Golgi cis cisterna, chemokine receptor activity, myosin heavy chain binding, biomineral tissue development, ER to Golgi vesicle-mediated transport, growth factor binding, Golgi vesicle transport, cis-Golgi network, peptide receptor activity
Signature 8	PRKCZ, TNFRSF9, PLA2G2D, RBP5, TTYH2, TMEM200C, TNFSF14, CLIP3, MMP9, EMID1, ITIH3, LTB, TRERF1, TMEM178B, KEL, ENPP2, PTGDS, LCNL1, CSF2RA, L1CAM	Extracellular region, tumor necrosis factor-mediated signaling pathway, response to tumor necrosis factor, cellular response to tumor necrosis factor, retinoid binding, isoprenoid binding, negative regulation of hydrolase activity, cytokine-mediated, signaling pathway, tumor necrosis factor receptor binding, negative regulation of peptidase activity

Table S4: Six gene signatures identified by the Dynamic Cut Tree and associated with poor prognosis.

Signature	Member genes	Gene Ontology
Signature 1	C1QA, C1QC, C1QB, DOCK7, IFI44L, IFI44, SORT1, FCGR1B, FCGR1A, SNX27, FCGR2A, FCGR3A, FCGR2C, FCGR3B, FCGR2B, ABL2, ARL8A, IFIT3, IFIT1, SCD, MXI1, CASP7, DHX32, SBF2, CAT, MS4A4A, CPT1A, PRCP, CASP5, SIK2, APLP2, CD163, PLBD1, EPS8, MGST1, KIF21A, GNS, LTA4H, OAS1, AQP9, TLN2, PDE8A, MT1G, CPNE2, ITGB3, SCPEP1, USP32, PSTPIP2, FECH, ADGRE1, GMFG, BLVRB, LILRA6, HK2, MARCO, SMIM25, IFNAR1, IFNGR2, ITSN1, RCAN1, DOP1B, MX1, APOBEC3H, DNAJC13, RNF13, RARRES1, PPM1L, APOD, SPP1, ELOVL6, IQGAP2, HSD17B4, SLC22A4, CYSTM1, PPARGC1B, TUBB2A, TUBB2B, AGPAT4, CD36, PDK4, NAMPT, KIAA1549, MSR1, CA2, ACO1, FRMD3, TMOD1, TXN, MAPKAP1, FCN1, VSIG4, DIAPH2	IgG binding, immune system process, immune response, neutrophil activation, immunoglobulin binding, leukocyte degranulation, neutrophil activation involved in immune response, neutrophil mediated immunity, neutrophil degranulation, cytoplasmic vesicle membrane
Signature 2	FAM41C, DIO1, HSP90B3P, CHRNB2, MROH9, FMO3, KMO, CHML, C10orf25, STOX1 RAG1, TULP3, RIMKLB, FAM234B, RAB3IP, MRS2P2, CDK8, PCDH9, DCUN1D2, LIN52, IQCH, NRG4, ASB9P1, PDZD9, ZKSCAN2, RPGRIP1L, PMFBP1, ZNF433, ZNF136, ZNF585B, ZNF473, ZNF615, ZNF702P, NLRP9, ZNF772, ZIK1, ZNF530, ZNF418, ZNF606, ZNF8, ZSCAN22, NLRC4, LYG1, TTC30B, GZF1, GCNT7, PAXBP1, SERPIND1, SUCLG2, LNP1, ZDHHC23, STXBP5L, TFDP2, LRRC34, CEP19, COQ2, WDR17, ADAMTS6, HOMER1, GLRA1, ERVFRD-1, ZNF391, ZNF165, ZKSCAN3 SCUBE3, EPM2A, ZNF713, SYP, ZC3H12B, MORC4	Cation binding, metal ion binding, RNA polymerase II-specific DNA-binding transcription factor activity, regulation of neurotransmitter secretion, DNA-binding transcription factor activity, transcription by RNA polymerase II, regulation of synaptic vesicle transport, regulation of synaptic vesicle exocytosis, DNA-templated transcription
Signature 3	MMACHC, ATP1A1.AS1, ARNT, CFAP126, GVQW3, INTS4, EP400P1, FAM161B, SNORD116.18, ADPGK, TIGD7, LONP2, KIAA0753, LINC00674, WDR7, ZNF177, ZNF439, ZNF844, ZNF788P, GPATCH1, ZNF461, ZNF850, ZNF45, ZNF226, ZNF227, ZNF577, ZNF649, ZNF836, ZNF808, ZNF701, ZNF28, ZNF320, ZNF160, ZNF845, ZNF525, ZNF765, ZNF761, ZNF813, ZSCAN5A, ZNF805, ZNF549, ZNF551, ZNF776, ZNF552, ZNF587B, ZNF587, ZNF814, ZNF417, PIGF, ALMS1, ANKRD36C, ANKRD36B, TIGD1, KIZ, CFAP44, MAP6D1, CPLANE1, ZNF354A, PHIP, CASP8AP2, FBXL4, AHI1, SGCE, PAXIP1.AS2, AGO2, NOL8, ZNF782, TOR1B, CXorf21	DNA-binding transcription factor activity, RNA polymerase II-specific, DNA-binding transcription factor activity, DNA binding, nucleic acid binding, transcription by RNA polymerase II, regulation of transcription by RNA polymerase II, RNA biosynthetic process, regulation of nucleic acid-templated transcription, regulation of RNA biosynthetic process, transcription, DNA-templated, mitotic cell cycle
Signature 4	DNAJC6, CHD1L, CD5L, KIF14, STK33, KIF18A, FOXM1, TROAP, TIMELESS, NUP107, GOLGA2P5, KNTC1, DIAPH3, DLGAP5, KIF23, TICRR, TEDC2, PALB2, RFWD3, WWOX, TLCD1, CDC6, TOP2A, BRCA1, EME1, BIRC5, ANKLE1, CCNE1, MTA3, MSH2, MSH6, PNPT1, FAM161A, ANAPC1P1, ANAPC1, POLR1B, KLHL23, CCDC150, AP1S3, MCM8, AURKA, DONSON, POLQ, TOPBP1, USP13, NUP155, NLN, TAF7, LSM11, POLH, MDN1, MTHFD1L, MAP3K4, SLC25A13, DNAJC2, PUS7, VIRMA, TBC1D31, KIF24, STRBP, XK	Regulation of mitotic cell cycle, cell cycle phase transition, mitotic cell cycle checkpoint, DNA metabolic process, mitotic cell cycle phase transition, DNA replication, mitotic DNA integrity checkpoint, negative regulation of mitotic cell cycle, regulation of cell cycle
Signature 5	ELOA, ACP6, POGZ, PDZD8, FAM204A, WDR11, IKZF5, DYRK4, ASB8, SLC35E3, CDADC1, PCCA, PIGH, TTLL5, RCN2, PDXDC1, RANBP10, FAM57A, PTRH2, PIAS2, DYM, ZCCHC2, ZNF561, ZNF709, ANKRD27, ZNF586, SUPT7L, DHX57, EHBP1, MAT2A, ZNF2, CCDC93, METAP1D, PTPRA, ATRN, PIK3R4, QDPR, NOCT, TBC1D7, PGBD1, SNORA5C, CSPP1, CCDC171, NAA35, ERCC6L2, ECPAS, ZNF79, MED27	Transferase complex, organelle membrane contact site, N-terminal protein amino acid modification, N-acetyltransferase activity, peptide N-acetyltransferase activity, acetyltransferase activity, N-acyltransferase activity, late endosome, catalytic complex, pigmentation
Signature 6	PARS2, CTH, POLR3C, CCT3, EPRS, SEPHS1, BCCIP, CKAP5, PRPF19, RTN3, ATP5F1B, SHMT2, TSFM, IPO5, PCID2, EEF2KMT, NDUFAB1, DCTPP1, NUP93, CSNK2A2, DDX19A, BLMH, PHB, DDX1, LRPPRC, GTF3C3, TMEM230, SAMM50, CCDC51, ATG3, RUVBL1, HADH, GAR1, HSPA9, GEMIN5, RPP40, CDC5L, TCP1, BZW2, RASA4CP, POLD2, IMMP2L, PTDSS1, CENPP	RNA localization, tRNA metabolic process, DNA metabolic process, protein-containing complex, cellular component assembly, chromosome, nucleoid, mitochondrial nucleoid, RNA transport, protein-containing complex assembly

Table S5: Univariate analyses of gene signatures identified by the pre-defined cutoff clustering method and the Dynamic Cut Tree method on the training and testing sets.

	**Pre-defined cutoff clustering method**				**Dynamic Cut Tree method**			
	**Training (n = 502)**		**Testing (n = 216)**		**Training (n = 502)**		**Testing (n = 216)**	
**Gene signatures**	**HR (95% CI)**	**p-value**	**HR (95% CI)**	**p-value**	**HR (95% CI)**	**p-value**	**HR (95% CI)**	**p-value**
Favorable 1	0.69 (0.56, 0.86)	0.00083	0.68 (0.5, 0.93)	0.015	0.66 (0.56, 0.78)	8.90×10^{-7}	0.77 (0.61, 0.97)	0.025
Favorable 2	0.73 (0.60, 0.88)	0.0012	0.72 (0.57, 0.90)	0.0038	0.54 (0.43, 0.68)	1.87×10^{-7}	0.63 (0.43, 0.91)	0.015
Favorable 3	0.77 (0.66, 0.90)	0.0014	0.77 (0.62, 0.95)	0.016	0.54 (0.40, 0.72)	4.67×10^{-5}	0.65 (0.44, 0.97)	0.032
Favorable 4	0.66 (0.50, 0.87)	0.0029	0.69 (0.48, 1.0)	0.051	0.64 (0.51, 0.80)	0.00014	0.66 (0.48, 0.91)	0.011
Favorable 5					0.59 (0.48, 0.73)	8.51×10^{-7}	0.60 (0.46, 0.78)	0.00014
Favorable 6					0.69 (0.58, 0.82)	3.34×10^{-5}	0.72 (0.57, 0.90)	0.005
Favorable 7					0.64 (0.52, 0.80)	6.20×10^{-5}	0.74 (0.55, 0.99)	0.042
Favorable 8					0.72 (0.61, 0.84)	6.75×10^{-5}	0.7 (0.56, 0.88)	0.002
Unfavorable 1	1.2 (1.1, 1.3)	0.0038	1.2 (1.0, 1.4)	0.025	1.8 (1.4, 2.3)	5.96×10^{-6}	1.5 (1.1, 2.2)	0.023
Unfavorable 2	2.2 (1.4, 3.4)	0.00058	2.1 (1.1, 3.9)	0.026	2.1 (1.5, 3.0)	1.01×10^{-5}	1.8 (1.1, 3.0)	0.016
Unfavorable 3	1.6 (1.2, 2.2)	0.0036	1.6 (1, 2.5)	0.04	2.1 (1.5, 3.1)	8.33×10^{-5}	1.6 (0.98, 2.7)	0.059
Unfavorable 4	1.5 (1.1, 1.9)	0.0048	1.5 (1, 2.2)	0.03	1.7 (1.3, 2.4)	0.00037	1.8 (1.1, 2.8)	0.010
Unfavorable 5	1.6 (1.2, 2.2)	0.0036	1.6 (0.98, 2.5)	0.058	5.6 (2.8, 11.0)	1.63×10^{-6}	3.6 (1.3, 9.6)	0.012
Unfavorable 6					1.9 (1.3, 2.6)	0.00037	1.8 (1.1, 3.1)	0.029

Abbreviations: CI, confidence interval; HR, hazard ratio.

Biological implications of gene-expression signatures

Table S6 summarizes the results from Kyoto Encyclopedia of Genes and Genomes (KEGG) pathway enrichment analyses of the gene-expression signatures using the WebGestalt tool [2]. We conducted differential expression tests with `edgeR` to determine differentially expressed genes in each gene-expression signature between ABC and GCB subtypes. We first chose genes according to a p-value cut-off of 5% and then applied a fold-change cut-off of 50%. Table S7 shows the differentially expressed genes between ABC and GCB according to this criteria, where GCB is the baseline category. To interpret the differential expression results in biological context, we conducted gene ontology (GO) enrichment analysis focusing on the ontology of biological process to determine the sets of genes that are up- or down-regulated between GCB and ABC. Table S8 shows the top 10 GO terms for the down- and up-regulated genes that are differentially expressed in each signature between GCB and ABC (Table S7). Table S9 highlights the important biological functions associated with the gene signatures of the survival model based on these analyses.

Figures S6 and S7 show the correlations between the gene signatures identified by the Dynamic Cut Tree method and the cell type abundances. Table S10 presents the correlation between the immune cell subtypes and gene signatures identified by the Dynamic Cut Tree method that had the most significant correlation. Figure S8 demonstrates the correlations between the risk score and immune cell fractions in the TME and tumor, and the corresponding p-values. We note that as the risk score increases, the survival experience worsens. In the absence of B-cells, CD4+ naive, CD4+ memory resting, regulatory and follicular helper T-cells and M0 macrophages were negatively associated with the risk score, implying that an increased infiltration of these cell subtypes is associated with improved survival outcome (all $p < 0.05$). In the presence of B-cells, we observed that CD4+ memory resting, follicular helper and regulatory T-cells and M0 macrophages were again significantly associated with favorable prognosis (all $p < 0.005$). Lastly, memory B cells were negatively correlated with the risk score ($p = 0.04$) whereas naive B-cells were positively correlated ($p = 0.04$), implying that increased naive B-cells are related with unfavorable prognosis.

In the presence of B-cells, we observed that CD4+ memory resting, follicular helper and regulatory T-cells and M0 macrophages were again significantly associated with favorable prognosis (all $p <$ 0.005, Figure S8). There was not an association between naive CD4+ T-cells and risk score when B-cells were added. Dendritic cells activated, neutrophils, eosinophils, mast cells resting, monocytes and M1/M2 macrophages were still significantly associated with unfavorable prognosis (all $p <$ 0.003). The negative correlation between the risk score and resting NK cells, plasma cells, CD4+ memory activated and gamma delta T-cells were stronger in the presence of B-cells (all $p < 0.08$). In contrast to case in the TME, activated NK cells demonstrated negative correlation with the risk score in the tumor ($p = 0.063$). Lastly, memory B cells were negatively correlated with the risk score ($p = 0.04$) whereas naive B-cells were positively correlated ($p = 0.04$), implying that increased

naive B-cells are related with unfavorable prognosis.

Figure S6: Heatmap showing the correlation between the gene signatures identified by the Dynamic Cut Tree method and the cell type abundances in the tumor microenvironment.

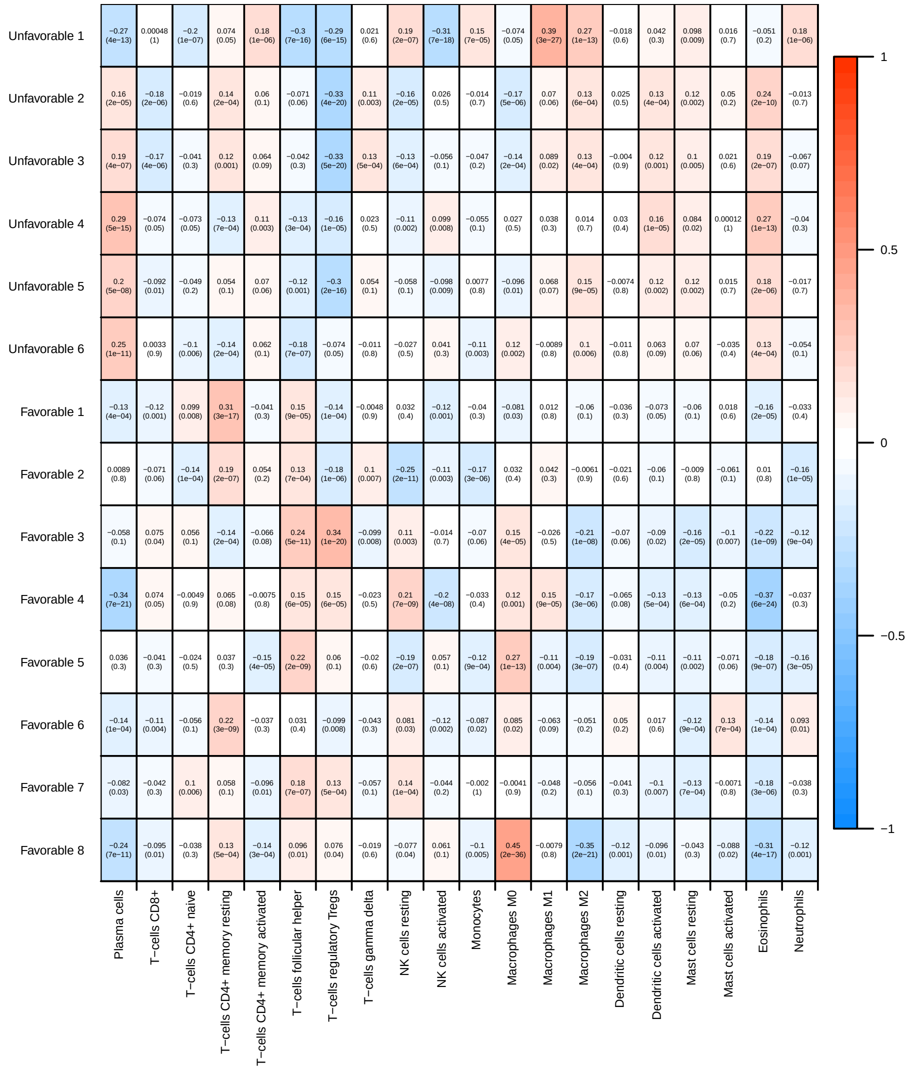

Figure S7: Heatmap showing the correlation between the gene signatures identified by the Dynamic Cut Tree method and the cell type abundances in the tumor including B-cells.

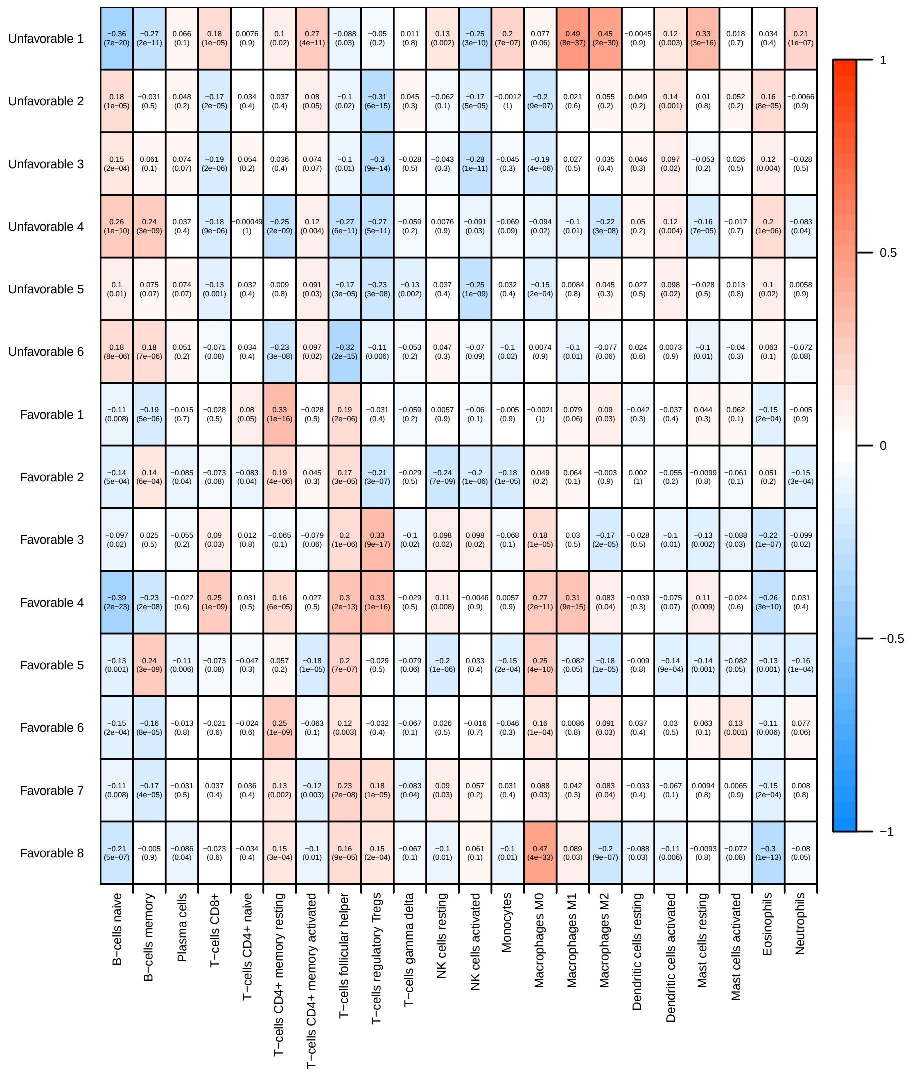

Table S6: KEGG pathway analyses results.

Pathway	Genes
**Good-prognosis gene signatures**	
**Signature 1**	
Antigen processing and presentation	TAPBP, HLA-B, CD4, HLA-F, HLA-E
Cell adhesion molecules	HLA-B, CD4, HLA-F, SELPLG, HLA-E
Endocytosis	HLA-B, LDLRAP1, HLA-F, HLA-E, ACAP1
Cytokine-cytokine receptor interaction	TNFRSF18, TNFRSF4, TNFRSF25, CSF1, PLEKHO2
Allograft rejection	HLA-B, HLA-F, HLA-E
Graft-versus-host disease	HLA-B, HLA-F, HLA-E
Type I diabetes mellitus	HLA-B, HLA-F, HLA-E
Autoimmune thyroid disease	HLA-B HLA-F, HLA-E
Viral myocarditis	HLA-B, HLA-F, HLA-E
Glycosaminoglycan biosynthesis - heparan sulfate	HS3ST3B1, HS3ST3A1
Primary immunodeficiency	CD4, JAK3
Phagosome	HLA-B, HLA-F, HLA-E
Hematopoietic cell lineage	CD4 , CSF1
Natural killer cell mediated cytotoxicity	HLA-B, HLA-E
Pathways in cancer	RALGDS , CEBPA
**Signature 2**	
Focal adhesion	PDGFB, FLT4, FLNB, SHC2
VEGF signaling pathway	SPHK1, SHC2
Glioma	PDGFB, SHC2
Arrhythmogenic right ventricular cardiomyopathy	CACNA1C , LMNA
Endocytosis	RAB11FIP5, IQSEC2, EHD2
MAPK signaling pathway	CACNA1C , PDGFB , FLNB
Hypertrophic cardiomyopathy	CACNA1C , LMNA
Dilated cardiomyopathy	CACNA1C, LMNA
Axon guidance	UNC5B , EPHA2
Calcium signaling pathway	SPHK1 , CACNA1C
Cytokine-cytokine receptor interaction	PDGFB, FLT4
**Signature 3**	
Gap junction	LPAR1, TJP1 , GJA1, EGFR
Regulation of actin cytoskeleton	GNG12, NCKAP1 MYLK, EGFR
Adherens junction	CTNND1, TJP1, EGFR
Focal adhesion	PARVA, MYLK, EGFR
Epithelial cell signaling in Helicobacter pylori infection	TJP1, EGFR
Cytokine-cytokine receptor interaction	IL1R1, IL6ST, EGFR
MAPK signaling pathway	IL1R1, GNG12, EGFR
Vascular smooth muscle contraction	CALD1, MYLK
Axon guidance	SEMA5A, ROBO1
Calcium signaling pathway	MYLK, EGFR
**Poor-prognosis gene signatures**	
**Signature 2**	
Proteasome	PSMD8, PSMC4, PSMB7
Metabolic pathways	NDUFA9, RPN2, GART, POLD3, TSTA3, ATP6V0D1, POLR3A
Protein export	SRPRB, SEC61A1
Purine metabolism	GART, POLD3, POLR3A
Vibrio cholerae infection	SEC61A1, ATP6V0D1
Aminoacyl-tRNA biosynthesis	FARSB, GARS
Pyrimidine metabolism	POLD3, POLR3A
Oxidative phosphorylation	NDUFA9, ATP6V0D1
Spliceosome	EIF4A3, SF3B3
Phagosome	SEC61A1, ATP6V0D1
Protein processing in endoplasmic reticulum	RPN2, SEC61A1
**Signature 3**	
Ribosome biogenesis in eukaryotes	CSNK2A1, WDR75, NVL, NMD3
RNA transport	NUP160, NMD3, XPOT, EIF2B1
Aminoacyl-tRNA biosynthesis	IARS
**Signature 4**	
Oocyte meiosis	AURKA, ANAPC1, CCNE1
Cell cycle	CDC6, ANAPC1, CCNE1
Colorectal cancer	BIRC5, MSH2
Pathways in cancer	BIRC5, CCNE, MSH2
Ubiquitin mediated proteolysis	ANAPC1, BRCA1
RNA transport	NUP155, NUP107
**Signature 5**	
Metabolic pathways	NDUFAB1, SHMT2, EPRS, HADH, PTDSS1, POLD2, SEPHS1
Wnt signaling pathway	RUVBL1, CSNK2A2
Spliceosome	PRPF19, CDC5L
RNA transport	GEMIN5, NUP93

Table S7: Differentially expressed genes between ABC and GCB subtypes in each gene signature.

Favorable 1			Favorable 2			Unfavorable 1			Unfavorable 2		
**Genes**	**log(FC)**	**p-value**	**Genes**	**log(FC)**	**p-value**	**Genes**	**log(FC)**	**p-value**	**Genes**	**log(FC)**	**p-value**
PCLO	-2.41	3.45×10^{-30}	MYBL1	-2.34	9.86×10^{-89}	PDK4	-1.00	7.50×10^{-12}	SERPIND1	-0.97	1.65×10^{-21}
NCAM1	-2.08	7.44×10^{-32}	MAPK10	-2.08	4.51×10^{-49}	TMOD1	-0.85	2.71×10^{-10}	KMO	-0.79	6.98×10^{-19}
LAMP3	-1.21	1.03×10^{-18}	SAMD12	-1.17	3.44×10^{-30}	APLP2	-0.78	1.25×10^{-39}	MROH9	0.61	0.00071
FGF13	-0.94	5.37×10^{-13}	NUP62CL	-1.03	1.45×10^{-10}	PSTPIP2	0.61	2.05×10^{-09}	ZNF391	0.71	5.93×10^{-09}
MMRN1	-0.93	9.98×10^{-12}	CACNB4	-0.91	9.72×10^{-10}	KIF21A	0.64	1.94×10^{-09}	HSP90B3P	0.75	1.65×10^{-21}
CXCL14	-0.93	1.29×10^{-10}	SLAMF1	-0.87	1.11×10^{-13}	FRMD3	0.64	5.86×10^{-10}	ADAMTS6	1.45	9.00×10^{-41}
SUSD5	-0.85	3.25×10^{-13}	PDGFD	-0.85	1.34×10^{-15}	FCGR1B	0.65	4.14×10^{-08}			
ARHGAP20	-0.76	1.56×10^{-10}	TEX9	-0.84	3.52×10^{-13}	PLBD1	0.67	3.76×10^{-20}			
GRIP1	-0.70	2.84×10^{-07}	NUDT10	-0.82	3.46×10^{-08}	C1QC	0.68	2.89×10^{-12}			
SLC1A2	-0.67	1.98×10^{-06}	DDI1	-0.74	9.25×10^{-09}	FCGR1A	0.70	2.03×10^{-09}			
SEMA3A	-0.66	6.39×10^{-13}	RRAS2	-0.66	4.21×10^{-18}	CD163	0.78	7.01×10^{-13}			
RELN	-0.60	1.60×10^{-05}	BPNT1	-0.66	4.16×10^{-26}	C1QA	0.82	4.88×10^{-15}			
NKD1	0.61	7.35×10^{-10}	FAM106CP	-0.61	1.46×10^{-07}	C1QB	0.95	7.79×10^{-17}			
IGHG2	0.62	4.63×10^{-06}	SLC1A1	-0.60	1.04×10^{-06}	MARCO	0.98	6.88×10^{-10}			
RIC3	0.64	8.34×10^{-05}	TMEM256	0.59	1.62×10^{-17}	CASP5	1.02	2.86×10^{-23}			
IGHG3	0.78	3.62×10^{-07}	TIGIT	0.61	1.30×10^{-10}	MT1G	1.18	3.65×10^{-10}			
SCN3A	0.84	5.68×10^{-05}	C22orf46	0.62	3.44×10^{-30}	ADGRE1	1.26	4.79×10^{-20}			
TRABD2A	0.90	9.89×10^{-17}	CIRBP	0.64	4.55×10^{-37}						
IGHV4-59	0.91	2.21×10^{-06}	CNTNAP2	0.64	0.00034						
COL4A5	0.93	1.70×10^{-07}	DDX17	0.68	6.65×10^{-78}						
HSF5	0.95	2.14×10^{-10}	EPHA4	0.73	2.10×10^{-07}						
IGKV1-5	1.07	7.11×10^{-07}	DNAH8	0.94	3.26×10^{-07}						
MAATS1	1.25	3.11×10^{-19}	IGSF10	1.03	1.05×10^{-13}						
IGHV3-7	1.27	2.73×10^{-10}									
IGHV3-21	1.39	1.29×10^{-10}									
HLF	2.77	6.44×10^{-45}									

FC: fold-change. p-values are adjusted for multiplicity errors by the Benjamini-Hochberg method.

Table S8: Gene ontology analysis of the differentially expressed genes between GCB and ABC.

	Down-regulated					Up-regulated				
**Gene signatures**	**GO Term**		**N**	**Down**	**p-value**	**GO Term**		**N**	**Up**	**p-value**
**Favorable 1**	GO:0030900	Forebrain development	4	4	0.001	GO:0045944	Positive regulation of transcription by RNA polymerase II	4	3	0.081
	GO:0021537	Telencephalon development	4	4	0.001	GO:0030111	Regulation of Wnt signaling pathway	4	3	0.081
	GO:0032990	Cell part morphogenesis	10	6	0.005	GO:0060070	Canonical Wnt signaling pathway	2	2	0.089
	GO:0048858	Cell projection morphogenesis	10	6	0.005	GO:0016311	Dephosphorylation	2	2	0.089
	GO:0048812	Neuron projection morphogenesis	10	6	0.005	GO:0001654	Eye development	2	2	0.089
	GO:0120039	Plasma membrane bounded cell projection morphogenesis	10	6	0.005	GO:0048592	Eye morphogenesis	2	2	0.089
	GO:0021795	Cerebral cortex cell migration	3	3	0.008	GO:0001754	Eye photoreceptor cell differentiation	2	2	0.089
	GO:0021987	Cerebral cortex development	3	3	0.008	GO:0060537	Muscle tissue development	2	2	0.089
	GO:0021885	Forebrain cell migration	3	3	0.008	GO:0030178	Negative regulation of Wnt signaling pathway	2	2	0.089
	GO:0007612	Learning	3	3	0.008	GO:0046530	Photoreceptor cell differentiation	2	2	0.089
**Favorable 2**	GO:0002250	Adaptive immune response	4	4	0.010	GO:0048699	Generation of neurons	9	7	0.016
	GO:0006955	Immune response	9	6	0.031	GO:0022008	Neurogenesis	9	7	0.016
	GO:0098655	Cation transmembrane transport	3	3	0.033	GO:0048513	Animal organ development	17	11	0.016
	GO:0002263	Cell activation involved in immune response	3	3	0.033	GO:0006996	Organelle organization	17	11	0.016
	GO:0051186	Cofactor metabolic process	3	3	0.033	GO:0048731	System development	26	15	0.018
	GO:0060047	Heart contraction	3	3	0.033	GO:0007399	Nervous system development	13	9	0.018
	GO:0003015	Heart process	3	3	0.033	GO:0007417	Central nervous system development	4	4	0.021
	GO:0002366	Leukocyte activation involved in immune response	3	3	0.033	GO:0051960	Regulation of nervous system development	4	4	0.021
	GO:0002275	Myeloid cell activation involved in immune response	3	3	0.033	GO:0050767	Regulation of neurogenesis	4	4	0.021
	GO:0002274	Myeloid leukocyte activation	3	3	0.033	GO:0006396	RNA processing	4	4	0.021
**Unfavorable 1**	GO:0065007	Biological regulation	63	28	0.003	GO:0050776	Regulation of immune response	13	9	0.001
	GO:0007264	Small GTPase mediated signal transduction	5	5	0.004	GO:0006955	Immune response	33	16	0.001
	GO:0009966	Regulation of signal transduction	19	12	0.005	GO:0002253	Activation of immune response	11	8	0.001
	GO:0035556	Intracellular signal transduction	15	10	0.006	GO:0050778	Positive regulation of immune response	11	8	0.001
	GO:0048519	Negative regulation of biological process	35	18	0.008	GO:0002250	Adaptive immune response	7	6	0.001
	GO:0065008	Regulation of biological quality	30	16	0.009	GO:0051604	Protein maturation	4	4	0.005
	GO:0032501	Multicellular organismal process	41	20	0.010	GO:0016485	Protein processing	4	4	0.005
	GO:0044237	Cellular metabolic process	44	21	0.011	GO:0030449	Regulation of complement activation	4	4	0.005
	GO:0050794	Regulation of cellular process	53	24	0.012	GO:2000257	Regulation of protein activation cascade	4	4	0.005
	GO:0007265	Ras protein signal transduction	4	4	0.013	GO:1903317	Regulation of protein maturation	4	4	0.005
**Unfavorable 2**	GO:0009968	Negative regulation of signal transduction	3	3	0.028	GO:0006357	Regulation of transcription by RNA polymerase II	19	6	0.174
	GO:0008277	Regulation of G protein-coupled receptor signaling pathway	3	3	0.028	GO:0006366	Transcription by RNA polymerase II	20	6	0.214
	GO:0008589	Regulation of smoothened signaling pathway	3	3	0.028	GO:0006026	Aminoglycan catabolic process	1	1	0.214
	GO:0007224	Smoothened signaling pathway	3	3	0.028	GO:0006022	Aminoglycan metabolic process	1	1	0.214
	GO:0050896	Response to stimulus	20	10	0.035	GO:0060249	Anatomical structure homeostasis	1	1	0.214
	GO:0060429	Epithelium development	4	3	0.089	GO:0035904	Aorta development	1	1	0.214
	GO:0002009	Morphogenesis of an epithelium	4	3	0.089	GO:0060840	Artery development	1	1	0.214
	GO:0048585	Negative regulation of response to stimulus	4	3	0.089	GO:0048148	Behavioral response to cocaine	1	1	0.214
	GO:1901566	Organonitrogen compound biosynthetic process	4	3	0.089	GO:0001568	Blood vessel development	1	1	0.214
	GO:0055114	Oxidation-reduction process	4	3	0.089	GO:0070509	Calcium ion import	1	1	0.214

GO: Gene ontology; N: number of genes in the GO term; Down/Up: number of down-/up-regulated differentially expressed genes. p-value represents the over-representation of GO term in down-/up-regulated genes.

Table S9: Gene ontology of the differentially expressed genes within the survival signatures in the comparison of ABC vs. GCB.

**Gene signature**	**Direction**	**GO enrichment analysis**
Unfavorable 1	Up	Humoral immune response<br>Acute immune response
	Down	Ras signal transduction<br>Cellular metabolism
Unfavorable 2	Up	Cardiovascular system development and angiogenesis<br>Dendritic spine maintenance<br>Cell wall development and organization
	Down	Tissue morphogenesis and epithelium development<br>Neurogenesis and neural tube development<br>Embryonic development
Favorable 1	Up	Wnt signaling<br>Visual system development<br>Sensory system development<br>Hematopoiesis
	Down	Nervous system development<br>Cognition development
Favorable 2	Up	Neurogenesis<br>Organogenesis
	Down	Immune response, circulation and cell activation<br>Adaptive immune response

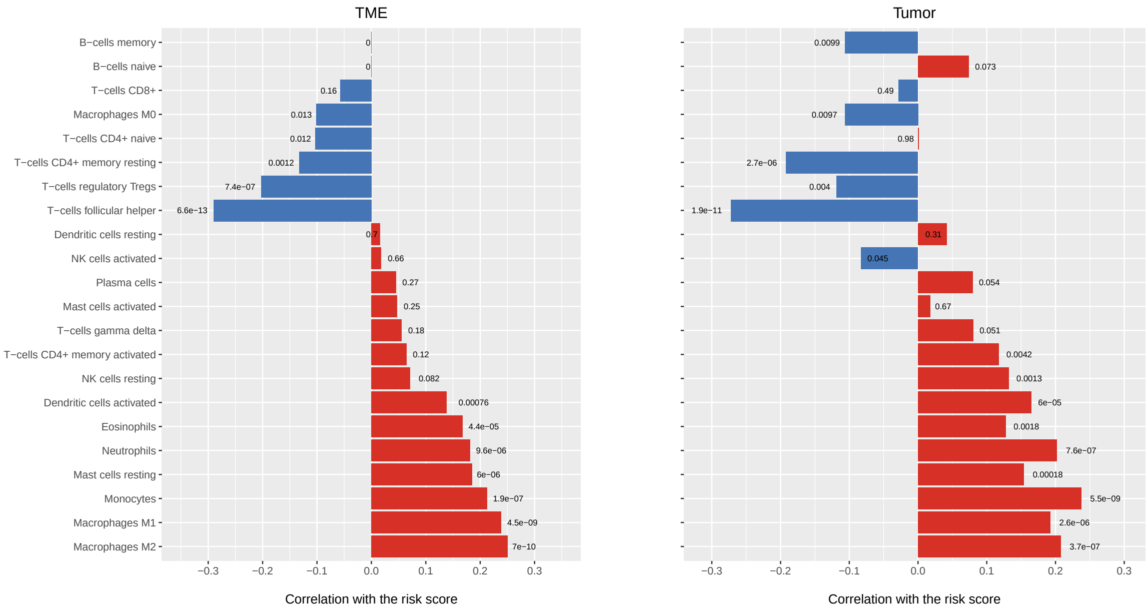

Figure S8: The association between the survival-predictor score and the immune cell fractions.

Table S10: Correlation between the gene signatures detected by the Dynamic Cut Tree method and the tumor-infiltrating cells in the TME and tumor.

	TME			Tumor		
**Immune cells**	**Gene signature**	**Correlation**	**p-value**	**Gene signature**	**Correlation**	**p-value**
**B-cells memory**				Unfavorable 1	-0.27	2.19×10^{-11}
**B-cells naive**				Favorable 4	-0.39	1.98×10^{-23}
**T-cells CD8+**	Unfavorable 2	-0.18	1.69×10^{-6}	Favorable 4	0.25	1.09×10^{-9}
**T-cells CD4+ naive**	Unfavorable 1	-0.20	1.03×10^{-7}	Favorable 2	-0.08	4.47×10^{-2}
**Macrophages M0**	Favorable 8	0.45	1.94×10^{-36}	Favorable 8	0.47	4.11×10^{-33}
**T-cells CD4+ memory resting**	Favorable 1	0.31	3.42×10^{-17}	Favorable 1	0.33	1.38×10^{-16}
**T-cells regulatory Tregs**	Favorable 3	0.34	1.47×10^{-20}	Favorable 3	0.33	9.09×10^{-17}
**T-cells follicular helper**	Unfavorable 1	-0.30	7.09×10^{-16}	Unfavorable 6	-0.32	1.65×10^{-15}
**Dendritic cells resting**	Favorable 8	-0.12	1.31×10^{-3}	Favorable 8	-0.09	3.34×10^{-2}
**Mast cells activated**	Favorable 6	0.13	7.17×10^{-4}	Favorable 6	0.13	1.07×10^{-3}
**NK cells activated**	Unfavorable 1	-0.31	6.85×10^{-18}	Unfavorable 3	-0.28	1.01×10^{-11}
**NK cells resting**	Favorable 2	-0.25	1.69×10^{-11}	Favorable 2	-0.24	7.15×10^{-9}
**T-cells CD4+ memory activated**	Unfavorable 1	0.18	1.38×10^{-6}	Unfavorable 1	0.27	4.37×10^{-11}
**Plasma cells**	Favorable 4	-0.34	6.50×10^{-21}	Favorable 5	-0.11	6.41×10^{-3}
**T-cells gamma delta**	Unfavorable 3	0.13	5.38×10^{-4}	Unfavorable 5	-0.13	1.95×10^{-3}
**Dendritic cells activated**	Unfavorable 4	0.16	1.28×10^{-5}	Favorable 5	-0.14	9.02×10^{-4}
**Neutrophils**	Unfavorable 1	0.18	1.05×10^{-6}	Unfavorable 1	0.21	1.46×10^{-7}
**Eosinophils**	Favorable 4	-0.37	6.05×10^{-24}	Favorable 8	-0.30	1.19×10^{-13}
**Mast cells resting**	Favorable 3	-0.16	1.56×10^{-5}	Unfavorable 1	0.33	2.72×10^{-16}
**Monocytes**	Favorable 2	-0.17	3.20×10^{-6}	Unfavorable 1	0.20	6.80×10^{-7}
**Macrophages M1**	Unfavorable 1	0.39	2.74×10^{-27}	Unfavorable 1	0.49	7.94×10^{-37}
**Macrophages M2**	Favorable 8	-0.35	2.18×10^{-21}	Unfavorable 1	0.45	1.84×10^{-30}

References

[1] Langfelder P, Zhang B, Horvath S. Defining clusters from a hierarchical cluster tree: the Dynamic Tree Cut package for R. Bioinformatics. 2007;24(5):719–720.

[2] Liao Y, Wang J, Jaehnig EJ, Shi Z, Zhang B. WebGestalt 2019: gene set analysis toolkit with revamped UIs and APIs. Nucleic acids research. 2019;47(W1):W199–W205.
